# Nutrition as Modulator of Oxidative Stress in Cancer Prevention and Treatment

**DOI:** 10.3390/ijms27157047

**Published:** 2026-08-06

**Authors:** Luciana Vallorani, Tatiana Balashova, Cesare Cremon, Fabio Vivarelli, Donatella Canistro, Camilla Morosini, Moreno Paolini, Alessandra Rossi

**Affiliations:** 1 Department of Biomolecular Sciences, University of Urbino Carlo Bo, 61029 Urbino, Italy; luciana.vallorani@uniurb.it; 2 School of Culinary Arts and Food Technology, Technological University Dublin (TUD), D07 EWV4 Dublin, Ireland; tibalashova@gmail.com; 3 IRCCS Azienda Ospedaliero, Universitaria di Bologna, 40138 Bologna, Italy; cesare.cremon@aosp.bo.it; 4 Department of Medical and Surgical Sciences, University of Bologna, 40138 Bologna, Italy; 5 Department of Pharmacy and Biotechnology, University of Bologna, 40126 Bologna, Italy; fabio.vivarelli3@unibo.it (F.V.); donatella.canistro@unibo.it (D.C.); camilla.morosini2@unibo.it (C.M.); moreno.paolini@unibo.it (M.P.)

**Keywords:** cancer prevention, oxidative stress, antioxidants, chemotherapy, ketogenic diet, fasting, calorie restriction, colorectal cancer, breast cancer, lung cancer, ovarian cancer, glioblastoma

## Abstract

Cancer remains a leading global cause of morbidity and mortality, with oxidative stress playing a central role in its pathogenesis, progression, and response to therapy. Nutrition has emerged as a modifiable factor capable of influencing redox homeostasis, thereby contributing to both cancer prevention and therapeutic outcomes. This narrative review summarizes current evidence on dietary patterns, specific nutrients, and bioactive compounds that modulate oxidative stress and are associated with reduced cancer risk and improved response to treatment. A literature search was conducted using PubMed, Scopus, and Google Scholar, covering the period from 2002 to 2025 to capture contemporary dietary approaches and clinical evidence. Eligible studies addressed nutrient compounds, dietary strategies, obesity, gut microbiota, conventional cancer therapies, and clinical outcomes, with particular emphasis on mechanisms related to oxidative stress, inflammation, and immune modulation. Epidemiological evidence consistently supports the protective role of plant-based dietary patterns and reduced intake of red and processed meat, partly due to their antioxidant and anti-inflammatory properties. Nutrients such as dietary fiber, polyphenols, and omega-3 fatty acids are associated with decreased oxidative damage, improved immune responses, and enhanced therapeutic efficacy across multiple tumor types, including colorectal, breast, lung, and ovarian cancers, as well as glioblastoma. Emerging data also suggest that dietary interventions, including ketogenic diets and fasting, may influence tumor metabolism and redox balance, potentially increasing sensitivity to conventional therapies. The gut microbiota has been identified as a key mediator linking diet, oxidative stress, and cancer-related pathways. Although current evidence supports the role of nutritional strategies in targeting oxidative stress for cancer prevention and treatment, further large-scale randomized clinical trials are required to clarify their impact on survival and treatment efficacy. The integration of nutritional counseling into oncology practice represents a cost-effective, accessible, and patient-centered approach with the potential to modulate oxidative stress and improve clinical outcomes.

## 1. Introduction

Dietary and lifestyle factors are recognized as major determinants of cancer risk. Indeed, the development of several malignancies, including colorectal cancer (CRC), breast cancer, ovarian cancer, and lung cancer, has been strongly associated with nutritional habits [1]. In particular, imbalanced diets can disrupt cellular redox homeostasis, leading to excessive production of reactive oxygen species (ROS) and impaired antioxidant defenses. This condition promotes oxidative damage to DNA, lipids, and proteins, thereby contributing to genomic instability, aberrant cell signaling, and tumor initiation and progression [2,3]. A high intake of red and processed meats has been linked to increased oxidative stress, partly due to the presence of heme iron, which can catalyze the formation of ROS through Fenton-like reactions. Additionally, high-temperature cooking methods such as grilling or frying can also lead to the formation of pro-oxidant compounds, including heterocyclic amines (HCAs) and polycyclic aromatic hydrocarbons (PAHs), further contributing to oxidative damage and carcinogenesis [4,5]. Moreover, variations in the levels of certain nutrients, including vitamins, trace elements and dietary fat, may also contribute to the establishment of a cancer-promoting environment [6].

Considerable research has investigated the role of nutrients as adjunctive strategies in the treatment of cancer. Conventional anticancer therapies particularly chemotherapy, remain the cornerstone of cancer treatment. However, chemotherapy is frequently associated with severe adverse effects including anemia, nausea, organ damage and neutropenic enterocolitis. Moreover, although it is highly effective for certain tumors, its therapeutic efficacy remains limited in other cancer types [7]. Recent evidence suggests that combining dietary strategies with chemotherapy may enhance treatment efficacy [8]. Preclinical and clinical studies have also demonstrated that nutritional approaches such as fasting or fasting-mimicking diets may promote differential stress resistance, protecting normal cells from oxidative damage while sensitizing cancer cells to treatment-induced cytotoxicity [9].

## 2. Literature Selection

This narrative review was written by examining peer-reviewed literature relevant to bioactive compounds, nutritional approaches in cancer prevention and therapy. A broad non-systematic search of scientific literature was conducted using PubMed and Scopus, combined with Google Search to identify additional evidence. This search focused on publications from 2002 to 2025. This time frame was chosen to capture contemporary evidence generated in the era of modern nutritional approaches and medicine research. The search was conducted using the following key words: “cancer”, “diet”, “nutrition”, “chemotherapy”, “cancer treatment”, “fasting”, “short-term fasting”, “intermittent fasting”, “ketogenic diet”, “caloric restriction”, “oxidative stress”, “Mediterranean diet”, “antioxidant supplements”, “nutrients”, “polyphenols”, “bioactive compounds”, “reactive oxygen species”, “clinical trials”. Inclusion criteria were as follows: (1) studies involving adult male and female participants; (2) studies investigating fasting, caloric restriction, or ketogenic diets during cancer treatment; (3) studies evaluating bioactive compounds in cancer prevention; and (4) studies reporting treatment-related outcomes, patient-reported outcomes, or changes in circulating biomarkers associated with insulin sensitivity, cancer-promoting growth factors, or oxidative stress. The following exclusion criteria were applied: (1) fasting performed as a religious practice; (2) studies conducted exclusively in pediatric or veterinary populations; (3) studies not related to cancer prevention or treatment; and (4) conference abstracts, editorials, non-peer-reviewed publications, and unpublished data. Original studies, systematic reviews, and meta-analyses published in English were eligible for inclusion when they provided relevant evidence on dietary interventions and cancer. Randomized controlled trials in humans were regarded as the primary source of evidence for clinical conclusions, whereas animal and in vitro studies were used mainly to explain biological mechanisms and generate hypotheses. Approximately 240 original studies, systematic reviews, and meta-analyses were reviewed.

In accordance with the narrative format, no formal risk of bias assessment was performed.

In this section, we synthesized and reported in a narrative format, according to the aims of a non-systematic review, the evidence identified in the literature search. These studies were organized in two major paragraphs describing the bioactive compounds and diet strategies relevant for cancer prevention and as adjuvant in conventional cancer treatments.

## 3. Nutritional Approaches in Cancer Prevention

Proper nutrition can contribute to preventing the onset of some tumors. Among the modifiable factors influencing cancer risk and mortality, diet is considered one of the most important. The latest report from the World Cancer Research Fund International (WCRF) and the American Institute for Cancer Research (AICR) estimates that approximately 29% of cases of the 13 most common cancers could be prevented through healthy lifestyle habits, particularly by maintaining a healthy body weight and consuming a balanced diet. Therefore, nutrition plays a fundamental role in the life of each individual. In the following sections, we summarize the effects of dietary patterns, specific nutrients and bioactive compounds on the onset of several types of cancer [4,5] (Table 1).

### 3.1. Whole-Food Source and Isolated High-Dose Supplementations

The distinction between whole-food source and high-dose supplementations is a fundamental concept in nutritional science. Whole-foods provide a complex matrix of vitamins, minerals, fiber, and phytochemicals that interact synergistically to influence metabolism, inflammation, and oxidative stress. Nutrients affect each other’s absorption, such as copper-zinc and manganese-iron [39]. Dietary bioactive compounds should not be regarded as universally protective or toxic molecules. Rather, their biological activity reflects a dynamic balance between cytoprotective redox regulation and oxidative signaling, which is influenced by dose, bioavailability, metabolic status, and disease context. This duality may partly explain the discrepancy between promising preclinical findings and the inconsistent results obtained in large randomized clinical trials, highlighting the need for personalized nutritional interventions based on individual redox status rather than indiscriminate antioxidant supplementation. Vitamin C represents a paradigmatic example of dual redox behavior. At nutritional concentrations, ascorbate maintains glutathione homeostasis, regenerates vitamin E, and scavenges reactive oxygen and nitrogen species. In contrast, pharmacological concentrations achieved by intravenous administration generate extracellular hydrogen peroxide through iron-dependent Fenton chemistry, thereby exerting selective pro-oxidant cytotoxicity against cancer cells while largely sparing normal tissues [39,40]. In contrast, isolated nutrients administered at pharmacological doses often fail to reproduce the health benefits associated with food-based dietary patterns and, in some cases, may even exert adverse effects. Preliminary evidence comparing consumption of fish with n-3 PUFA supplements suggests that consumption of the whole-food may deliver additional benefits not obtained with the single nutrient supplementation [41,42]. Through the generation of specialized pro-resolving mediators, omega-3 fatty acids attenuate chronic inflammation, inhibit NF-κB signaling, and indirectly reduce oxidative stress [40]. However, because of their high degree of unsaturation, they are highly susceptible to lipid peroxidation. Under conditions of increased oxidative stress, this property may facilitate ferroptosis, an iron-dependent form of regulated cell death currently being explored as a therapeutic vulnerability in several malignancies [43]. Several studies reported controversial data about antioxidant supplements [44,45]. Free radicals may play dual roles: free radicals in moderate concentrations are essential mediators of reactions by which unwanted cells are deleted from the body. However, excessive antioxidants might interfere with some essential defensive mechanisms of our organism [46]. It has been shown that antioxidant supplements seem to increase mortality by about 4% [47]. In a meta-analysis conducted by de Oliveira, it was shown that vitamin E consumption was inversely associated with breast cancer (BC) recurrence, although no association was found for vitamin E consumption and BC mortality [48]. It was also evidenced that supplementation of antioxidant N-acetylcysteine (NAC) and vitamin E markedly increases tumor progression and reduces survival in a mouse model of lung cancer. Despite the well-documented role of vitamin E in interrupting lipid peroxidation chain reactions and preserving membrane integrity; excessive supplementation may suppress physiological ROS signaling required for immune surveillance and disrupt the ROS-p53 axis [49]. Excessive antioxidant supplementation may interfere with the antitumor immune surveillance by altering the physiological ROS signaling. ROS are essential mediators of immune cell activation, including T-cell receptor signaling, dendritic cell maturation, and natural killer cell function. Therefore, excessive ROS scavenging may impair immune effector mechanisms and create a more permissive environment for tumor immune escape [50,51]. Polyphenols, carotenoids, and essential minerals represent key dietary bioactive compounds involved in the regulation of oxidative stress and cancer biology [6]. Under physiological conditions, these compounds generally exert antioxidant effects by scavenging reactive oxygen and nitrogen species, enhancing endogenous antioxidant defenses through activation of the Nrf2 pathway, and preserving mitochondrial function and cellular redox homeostasis [52,53]. However, increasing evidence indicates that their biological activity is highly context-dependent. Conversely, at higher concentrations or in the presence of iron and copper, several polyphenols—including quercetin, epigallocatechin gallate (EGCG), and resveratrol—undergo auto-oxidation or redox cycling, generating hydrogen peroxide and semiquinone radicals that promote oxidative stress and apoptosis in malignant cells. This pro-oxidant activity has been proposed as one of the mechanisms underlying the selective cytotoxicity of several polyphenols toward cancer cells [54,55,56]. Similarly, while minerals such as selenium and zinc are essential cofactors of antioxidant enzymes, excessive selenium intake may trigger pro-oxidant mechanisms that contribute to cancer cell death but may also increase toxicity [57]. Therefore, the dual antioxidant/pro-oxidant properties of these bioactive compounds should be carefully considered when evaluating their potential role as adjunctive strategies in cancer prevention and treatment, as their effects depend on dose, tumor type, and the cellular redox environment. Dietary bioactive compounds should not be regarded as universally antioxidant or pro-oxidant molecules. Rather, their biological activity reflects a dynamic balance between cytoprotective redox regulation and oxidative signaling, which is influenced by dose, bioavailability, metabolic status, and disease context. This duality may partly explain the discrepancy between promising preclinical findings and the inconsistent results obtained in large randomized clinical trials, highlighting the need for personalized nutritional interventions based on individual redox status rather than indiscriminate antioxidant supplementation.

Consequently, current evidence supports obtaining bioactive compounds primarily through a balanced diet rather than through a high-dose supplementation, except when clinically indicated.

### 3.2. Prostate Cancer

Prostate cancer (PCa) is the most frequently diagnosed malignancy among men and represents a major cause of cancer-related mortality worldwide [58]. Increasing evidence indicates that metabolic disorders, including obesity, insulin resistance, and hyperglycemia, contribute to PCa initiation and progression toward more aggressive disease phenotypes. Considerable research has addressed whether factors that affect the inflammation are associated with prostate cancer risk. With the exception of obesity, which is associated with an increased inflammation and higher risks of the high-grade PCa and death, studies on lifestyle factors correlated to the reduced inflammation, including use of aspirin and nonsteroidal anti-inflammatory drugs and statins have been inconsistent [59,60,61]. From a dietary standpoint, there has been considerable interest in the association of long-chain omega-3 polyunsaturated fatty acids (n-3 PUFA), which is a biomarker of the usual ω-3 fatty acid intake, with PC incidence. In 2011, Brasky and colleagues published a case–cohort study in which men in the highest quartile of plasma n-3 PUFA levels were found to have an increased risk of low grade, high grade, and total PC when compared to men in the lowest quartile [62]. In the large prospective trial SELECT, high plasma phospholipid concentrations of long-chain ω-3 PUFA were associated with statistically significant increases in the PCa risk. These associations were similar for low- and high-grade disease and for eicosapentaenoic acid (EPA) and docosahexaenoic acid (DHA), which are anti-inflammatory, metabolically interrelated to ω-3 fatty acids derived from oily fish and fish oil supplements [63]. DHA, obtained through dietary intake or supplementation, accumulates in prostate tissue and may exert antitumor effects at both the tissue and cellular levels. At the tissue level, DHA seems to influence stromal components, such as the inhibition of cancer-associated fibroblast differentiation and resolution of inflammation, which generates a microenvironment favorable to PCa initiation and progression. At the cellular level, DHA suppresses PCa cell growth through multiple mechanisms that depend on androgen receptor (AR) status. In AR-positive cells, DHA promotes proteasomal degradation of the AR, leading to downregulation of androgen-responsive genes and sterol regulatory element-binding proteins (SREBPs), thereby reducing fatty acid synthase (FASN) expression and lipogenesis. Also, DHA dysregulates mitochondria and induces ROS overproduction, which is closely related to cell death. Increase in DHA uptake is harmful to the cells because the rise in unsaturation status in their membrane sensitizes them to oxidative damage, but also changes membrane properties related to cell signaling, lipid packing, fluidity, and permeability [64].

Subsequent meta-analyses have found small and non-significant direct relationships between circulating n-3 PUFA levels and total PCa risk. A meta-analysis conducted by Conrad et al., provided no strong evidence of a protective association of fish consumption with prostate cancer incidence but showed a significant 63% reduction in prostate cancer–specific mortality, by preventing metastatic disease [65]. Moreover, Farrel et al. found no evidence that consuming n-3 PUFA-rich fish or using fish oil supplements affects the risk of PC [66]. Therefore, the role of DHA in prostate carcinogenesis remains inconclusive, and its contribution, as suggested by epidemiological evidence, requires further investigation through well-designed studies [67].

Preclinical studies conducted on rats showed that the caloric restriction (CR) may represent a useful intervention in aging, as it could prevent age-associated diseases, such as prostate hyperplasia, and extend longevity [68]. The authors supported previous evidence demonstrating that the CR has a pleiotropic effect modulating the main hallmarks of aging, such as immune inflammation and oxidative stress, which drive the tissue remodeling of the prostate while aging that notoriously increases the risk of PCa development [68]. In a prospective cohort of 43.435 Japanese men, Kurahashi et al. reported that higher intakes of dairy products, milk, and yogurt were associated with an increased risk of prostate cancer. Intake of cheese was not statistically associated with total PCa nor the calcium intake [33]. Current evidence suggests a modest positive association between high dairy (particularly milk) consumption and prostate cancer risk [33]. However, the evidence is limited to observational studies, the magnitude of the association is small, and causality has not been established.

Lycopene is a tetra-terpene from the carotenoid family, which is found in tomatoes and in red fruits and vegetables, such as red carrots, watermelons, strawberries, cherries, pomegranates, blood oranges, and papayas, and is responsible for reducing the risk of various cancers, particularly PCa. Intestinal absorption and, hence, the bioavailability of lycopene is improved by fats and by cooking of foods that contain it, for example, by cooking the tomato sauce. A case–control study conducted within the Physicians’ Health Study had already shown that the risk for aggressive PCa was significantly reduced in subjects with high concentration of lycopene [34]. Particularly, it has been shown a significant association between high plasma levels of lycopene and a strong reduction in aggressive PCa, a finding that was subsequently confirmed by other prospective studies, supporting a potential protective role of lycopene against PCa progression [35].

A pilot study investigated the effects of the MD on fatty acid composition, inflammatory biomarkers, and DNA damage in 20 men with PCa who adhered to the dietary intervention for three months. At the end of the study, participants showed a significant reduction in total saturated fatty acids, primarily due to decreased stearic acid intake, together with significant increases in circulating EPA and DHA levels. In addition, both the omega-6/omega-3 and arachidonic acid (AA)/EPA ratios were significantly reduced, indicating an improved fatty acid profile. Although no significant changes were observed in prostate-specific antigen (PSA) or C-reactive protein levels, greater adherence to the MD was significantly associated with reduced DNA damage, suggesting a potential protective effect on genomic stability [36].

Finally, in a pooled analysis of 15 prospective cohorts conducted by Sidahmed et al., weak nonsignificant associations were observed between total dietary fiber intake and risk of advanced forms of PCa, high-grade PC, and PC mortality. High dietary fiber intake from grains was associated with a modestly lower risk of advanced forms of PC and PC mortality [37].

Regarding dietary supplementation for PC prevention, it has attracted considerable interest over the past three decades, largely driven by preclinical evidence suggesting that antioxidant compounds, particularly selenium and vitamin E, might reduce oxidative DNA damage and inhibit prostate carcinogenesis. However, clinical evidence has largely failed to confirm these expectations, and current evidence does not support the routine use of vitamin E, selenium, or other antioxidant supplements for prostate cancer prevention. Contrary to the initial hypothesis, vitamin E supplementation was associated with a 17% increased risk of prostate cancer compared with placebo after extended follow-up [38] and the current clinical recommendations of The American Society of Clinical Oncology (ASCO), the European Society for Medical Oncology (ESMO), the World Cancer Research Fund/American Institute for Cancer Research (WCRF/AICR), and the National Cancer Institute (NCI) conclude that available evidence does not support the use of vitamin E, selenium, or other dietary supplements for PC prevention in the general population [38,69,70,71].

### 3.3. Colorectal Cancer

As the third most common cancer in men and the second most common cancer in women worldwide, colorectal cancer (CRC) represents a major public health concern. Its etiology is multifactorial, and accumulating evidence indicates that lifestyle factors, especially diet, significantly influence CRC risk. Therefore, maintaining a healthy dietary pattern may represent an effective strategy for CRC prevention and incidence reduction [4,5].

A recent systematic review and meta-analysis evaluated the association between dietary patterns and CRC risk [4,5]. The authors reported that adherence to a healthy dietary pattern—characterized by a high consumption of vegetables, fruits, whole grains, olive oil, fish, soy products, poultry, and low-fat dairy products—was associated with a reduced risk of CRC. In contrast, a Western dietary pattern, characterized by a high intake of red and processed meat, alcohol, refined grains, sweets, high-fat dairy products, butter, and high-fat gravies, together with a low consumption of fruits and vegetables, was associated with an increased risk of CRC [4]. The protective effect of fruits and vegetables against CRC has been attributed to their high content of potentially anticarcinogenic compounds, including dietary fiber, folate, and a variety of vitamins and minerals with antioxidant properties [4,12,14].

Multiple mechanisms are thought to mediate the influence of diet on colorectal carcinogenesis. These include direct modulation of immune function and inflammatory responses, as well as indirect effects mediated by overnutrition and obesity, both established risk factors for CRC. In addition, growing evidence indicates that the gut microbiota represents a key mediator linking dietary habits to colorectal cancer development [12,72]. Indeed, diet is likely the major driver of gut microbial composition and function [73]. Since the gut microbiome has emerged as a key determinant of essential human functions, including metabolism, immune regulation, colonization resistance, and response to drugs, its imbalance, a condition known as dysbiosis, is associated with a broad range of intestinal and extraintestinal disorders, including CRC [74,75]. Dysbiosis may contribute to tumor initiation and progression through mechanisms involving chronic inflammation, genotoxic metabolite production, and the modulation of epithelial barrier integrity [76]. Specific microbial taxa, such as *Fusobacterium nucleatum*, enterotoxigenic *Bacteroides fragilis*, and colibactin-producing *Escherichia coli*, have been implicated in tumor-promoting pathways, including the onset and progression of CRC [77]. Among these microbes, *Fusobacterium nucleatum* (Fn), an oral anaerobic bacterium frequently enriched in colorectal tumor tissue, promotes colorectal carcinogenesis through multiple interconnected mechanisms, including the secretion of virulence factors, the induction of chronic inflammation, immune evasion, and direct crosstalk with tumor cells. and it is considered not only a biomarker but also a potential driver of CRC initiation and progression [78]. However, considerable strain-to-strain variation in Fn genotyping and phenotyping features has been described and its role in CRC remains a matter of debate [79]. A recent genomic study by Zepeda-Rivera et al. demonstrated that CRC is preferentially associated with Fn subspecies animalis (Fna). The authors identified two genetically and epigenetically distinct Fna clades: Fna C1, which is primarily confined to the oral cavity, and Fna C2, which is selectively enriched in CRC tissues [80]. The authors demonstrated that oral administration of the Fna C2 clade in APC^min+/−^mouse models significantly increased the number of colonic adenomas compared with Fna C1 and control animals. Compared with Fna C1, the C2 clade exhibits enhanced greater resistance to pH-related stress and metabolic adaptations to the intestinal environment, promotes a pro-tumorigenic intestinal microenvironment through alterations in glutathione metabolism, oxidative stress, and inflammatory pathways [80]. These findings indicate that the association between Fn and CRC is strain-specific rather than a general feature of the species.

There is evidence that prebiotic fibers, including legumes, fruits, vegetables, and other fiber-rich foods, particularly whole grains affect the gut microbiome by promoting healthy conditions in the intestinal microbial ecosystem, known as eubiosis [81]. Dietary fiber exerts beneficial effects on intestinal health largely through its fermentation by the gut microbiota, leading to the production of short-chain fatty acids (SCFAs), including butyrate, propionate, and acetate [82]. These microbial metabolites play an important role in maintaining intestinal homeostasis by regulating immune and inflammatory responses and have been implicated in protection against CRC. Moreover, a balanced gut microbial community supports the production of other bioactive metabolites, such as bile acids and vitamins, which further contribute to intestinal immune regulation. Conversely, dysbiosis or inadequate dietary habits may reduce the production of these beneficial microbial metabolites, promoting chronic inflammation and creating a microenvironment that favors CRC development [12,15]. A recent meta-analysis suggested that a higher intake of citrus, apple, watermelon, and kiwifruit was negatively associated with CRC, reducing the risk by 9%. On the other hand, the intake of other types of fruit was not significantly associated with CRC risk [83]. Although the results are sometimes conflicting, physicians should emphasize to patients that eating a diet high in fruits, vegetables, and whole grains reduces CRC risk [84].

Epidemiologic evidence concerning the incidence of CRC and the consumption of dairy products is inconsistent. Given the paucity of data, further investigations are needed. However, yogurt has a potential anti-CRC effect, which seems to be due to its probiotic content [12,15]. These microorganisms are essential for the health of the microbiota, as they inhibit the adhesion of pathogenic bacteria to the intestinal epithelium, thereby reducing the risk of CRC development [85].

Fruits and vegetables are rich sources of antioxidants and other ROS scavengers that help maintain cellular redox homeostasis. By limiting oxidative stress, these bioactive compounds may reduce the risk of carcinogenesis, as excessive ROS production can induce DNA damage, gene mutations, genomic instability, chronic inflammation, and uncontrolled cell proliferation. Among the major dietary antioxidants, carotenoids—including β-carotene, a precursor of vitamin A, and lycopene—as well as vitamins C and E possess potent antioxidant and anti-inflammatory properties. In addition, selenium is an essential trace element required for the activity of antioxidant selenoenzymes that protect cells against ROS-induced oxidative damage [10].

Moreover, a diet rich in fruits (especially citruses, apples, pears, and grapes) and vegetables (especially tomatoes, shallots, and potatoes) constitutes a rich source of chalcones. These bioactive compounds may interfere with multiple stages of carcinogenesis, exerting protective effects from tumor initiation through promotion and progression to angiogenesis, invasion, and ultimately metastasis. Orlikova et al. reported that chalcones suppress cell cycle progression while promoting cell death, predominantly through the induction of apoptosis in transformed cells. The regulation of these processes is generally attributed to the ability of chalcones to modulate a complex network of inflammatory cell signaling pathways that largely contribute to CRC promotion [10]. Cruciferous vegetables (members of the Brassicaceae family, such as broccoli, Brussels sprouts, cabbage, cauliflower, kale, radishes, and turnips) also contain isothiocyanates that prevent an increase in CRC cells [86].

Foods rich in vitamin D, including eggs, fish, and mushrooms, may also contribute to CRC prevention. Vitamin D exerts its biological effects by binding to and activating the nuclear vitamin D receptor (VDR), thereby regulating the transcription of numerous genes involved in carcinogenesis. Through VDR-mediated signaling, vitamin D exhibits multiple anticancer activities, including the inhibition of cell proliferation, induction of cellular differentiation and apoptosis, suppression of angiogenesis, and modulation of inflammatory responses, all of which may contribute to reducing CRC risk [12]. In addition to its direct anticancer effects, accumulating evidence identifies vitamin D as an important regulator of immune homeostasis. This function may be particularly relevant in the context of CRC, as the colonic mucosa is continuously exposed to dietary chemicals, microbial metabolites, and bacterial carcinogens that can promote chronic inflammation and carcinogenesis [14].

Fish and eggs are also particularly rich in omega-3 fatty acids. Consumption of PUFAs promotes the growth of bacteria capable of producing butyrate and prevents gut microbiota dysregulation [87]. In particular, polyphenols from plant-based foods and omega-3 PUFAs demonstrate inhibitory activity against pathogenic microbial taxa, including Fn, thus promoting eubiosis [88]. Preliminary evidence from randomized controlled trials (RCTs) suggests that dietary intake of omega-3 fatty acids may be associated with a reduced risk of CRC [89].

In contrast, convincing evidence indicates that high consumption of red and processed meat is associated with an increased risk of CRC. The risk increases linearly with increasing meat intake [90]. Dietary patterns rich in red and processed meats, saturated fats, and highly refined carbohydrate-rich foods are linked to increased abundances of Fn and other proinflammatory species, which are likely involved in carcinogenesis processes [91]. Several biological mechanisms have been proposed to explain the association between red meat consumption and CRC. Red meat is a rich source of sulfur-containing amino acids and saturated fatty acids, while processed meat also contains inorganic sulfur compounds commonly used as preservatives. In addition, the heme iron present in red meat promotes oxidative stress, stimulates colonocyte proliferation, and enhances the endogenous formation of N-nitroso compounds (NOCs), recognized gastrointestinal carcinogens. Processed meats further contribute to NOC exposure through nitrates and nitrites added during preservation. In contrast, consumption of poultry and fish has been associated with a modest reduction in CRC risk. Thus, on the basis of current evidence, replacing red and processed meat with poultry or fish may therefore represent a reasonable dietary strategy for CRC prevention [4,11,12,92].

### 3.4. Breast Cancer

Breast cancer (BC) is the most frequently diagnosed malignancy among women worldwide, with its incidence continuing to rise in both high- and low-/middle-income countries. Observational studies indicate that lifelong adherence to regular physical activity and healthy dietary patterns is associated with a lower risk of both premenopausal and postmenopausal BC. Furthermore, maintaining a healthy body weight throughout adulthood appears to be an effective strategy for reducing the risk of postmenopausal BC [19]. In particular, cohort studies consistently link overweight, obesity, and adult weight gain to the risk for postmenopausal BC: women who gain 20 kg, or more, during adulthood, double their BC risk [18,19].

Obesity-related chronic inflammation is a major contributor to BC development and progression, primarily through the activation of inflammatory signaling pathways and metabolic dysregulation. Excess adipose tissue promotes the sustained production of pro-inflammatory cytokines, creating a tumor-promoting microenvironment that facilitates breast carcinogenesis. Among modifiable lifestyle factors, diet has long been recognized as an important determinant of BC risk and mortality. More recently, increasing attention has focused on the potential role of dietary advanced glycation end products (AGEs), whose high intake has been associated with enhanced oxidative stress and chronic inflammation, thereby potentially increasing the risk of BC [93,94,95].

Among the dietary patterns investigated, the Mediterranean diet (MD) has the strongest supporting evidence for BC prevention. Characterized by a high intake of legumes, whole grains, fruits, nuts, vegetables, and extra-virgin olive oil, moderate consumption of red wine, and limited intake of red meat, poultry, and dairy products, the MD has consistently been associated with a reduced risk of several chronic diseases, including BC [19,20]. Dietary modification is widely recognized as a primary strategy for cancer prevention and has been estimated to reduce overall cancer risk by approximately 30–50% [6]. In a hospital-based case–control study conducted in Italy and Switzerland, Turati et al. reported that greater adherence to the MD was associated with a modest but significant reduction in BC risk among Southern European women, a population in which this dietary pattern remains widely practiced. Similar inverse associations between adherence to the MD and BC risk have also been reported in several other epidemiological studies [20].

The potential protective effect of the MD against BC is supported by several biological mechanisms. This dietary pattern is rich in dietary fiber and antioxidant compounds, including flavonoids, vitamins, carotenoids, and squalene, the latter being particularly abundant in extra-virgin olive oil. These bioactive compounds may reduce BC risk by modulating endogenous estrogen levels, increasing circulating sex hormone-binding globulin concentrations, scavenging reactive oxygen species, limiting DNA damage, and attenuating oxidative stress [20].

The MD is also an important source of vitamins, including vitamin D_3_, folate, vitamin B_6_, and β-carotene, as well as bioactive dietary compounds such as piperine, sulforaphane, indole-3-carbinol, quercetin, epigallocatechin gallate (EGCG), and PUFAs. These micronutrients and phytochemicals have been shown to inhibit key processes involved in BC progression, including cell proliferation, invasion, angiogenesis, and metastasis [6,16,18].

Balanced vegetarian and vegan dietary patterns have also been associated with a reduced risk of BC. Their high dietary fiber content may contribute to this protective effect by decreasing the intestinal reabsorption of estrogens and androgens, thereby lowering their circulating concentrations. Soluble fiber appears to confer the greatest benefit, possibly through improvements in insulin sensitivity. Conversely, higher consumption of red meat has been associated with a modest increase in BC risk, with each additional 100 g consumed per day corresponding to an approximately 4% increase in risk [19]. A higher intake of fruit, vegetables and fiber and a moderate intake of soy/isoflavone were associated with beneficial outcomes in preventing breast cancer [96]. Soy-based foods are a major source of isoflavones, phytochemicals that exhibit both weak estrogenic and anti-estrogenic activities, in addition to several hormone-independent anticancer effects. Experimental studies have shown that these compounds inhibit the growth of BC cells, while epidemiological evidence suggests that lifelong consumption of moderate amounts of soy is associated with a lower risk of BC [96].

Furthermore, long-chain omega-3 fatty acids, such as alfa-linoleum acid (ALA), EPA and DHA, play very important roles [16,17]. Both Mediterranean and vegetarian diets bring good quantities of these molecules (fish and other marine sources, such as flaxseed, nuts, eggs, and olive oil). Increasing interest in the potential role of omega-3 fatty acid supplementation in cancer prevention has been driven by observations of a higher incidence of BC in Western populations, where dietary omega-3 fatty acid intake is relatively low. In contrast, there was a very low incidence of this condition in populations with high marine omega-3 fatty acid intake (Japan and natives of Alaska and Greenland) [17].

Higher dietary intake of EPA and DHA has also been associated with improved BC outcomes. In a large cohort of more than 3.000 women with early-stage BC, greater consumption of these marine-derived n-3 PUFAs was associated with a 25% reduction in BC recurrence and improved overall survival [17]. Consistent with these findings, higher intake of n-3 PUFAs has been inversely associated with both BC incidence and breast cancer-specific mortality. The protective effects of EPA and DHA are thought to be mediated through multiple mechanisms, including inhibition of tumor cell growth, induction of apoptosis, and suppression of angiogenesis [18].

### 3.5. Lung Cancer

Lung cancer (LC) remains one of the leading causes of cancer-related mortality worldwide, with overall survival remaining extremely poor despite advances in diagnosis and treatment. LC is a highly lethal disease characterized by a complex and multifactorial process of carcinogenesis and despite the arrival of targeted therapy and precision medicine, the outcome of this pathology is poor and disappointing [23,24,25,26].

Tobacco smoking remains the primary risk factor for LC. Although smoking cessation is the most effective strategy for reducing LC risk, nicotine dependence makes quitting particularly challenging for many individuals. Consequently, identifying safe and effective preventive approaches for smokers remains a public health priority, as prevention continues to represent the most effective strategy for reducing LC mortality [23,26].

Growing attention has been directed toward dietary and nutritional interventions to prevent this cancer. In particular, the evidence refers to diets rich in fruit and vegetables and MDs as nutritional approaches that could reduce the incidence of LC [23,24,25,26,27,28,97].

A meta-analysis by Yang et al. reported that higher consumption of vegetables and fruits was associated with a 16% lower risk of LC among current smokers. Furthermore, each additional 100 g/day of vegetable and fruit intake was associated with a 2% reduction in LC risk in this population [23]. Another meta-analysis confirmed these results and revealed that an 8–18% decrease in LC risk was associated with increased intake of fruits and vegetables. The analysis further stratified participants according to smoking status and demonstrated a significant dose–response relationship between fruit and vegetable intake and LC risk among current smokers. In contrast, this association was weaker and less consistent in former and never smokers [29]. The beneficial effects of fruits and vegetables on human health are attributed to nutrients and biologically active compounds, such as phytochemicals, vitamins, minerals, and fibers.

Since oxidative stress plays a central role in lung carcinogenesis, particularly among smokers, whose exposure to tobacco smoke promotes the generation of excessive ROS, which contribute to cancer initiation and progression, antioxidants have long been considered potential agents for LC prevention.

However, the relationship between antioxidants and lung cancer is more complex than initially thought. Due to their high metabolic activity, cancer cells themselves produce large quantities of ROS, and to survive in this hostile environment, both normal and malignant cells rely on sophisticated antioxidant defense systems that maintain redox homeostasis. Endogenous antioxidant enzymes, including catalases and peroxidases, are often overexpressed in cancer cells as a consequence of the constitutive activation of nuclear factor erythroid 2-related factor 2 (NRF2), the principal transcriptional regulator of cellular antioxidant defense [2]. Activation of the NRF2 pathway enhances the antioxidant capacity of tumor cells, allowing them to cope with oxidative stress and potentially promoting tumor survival and progression. The nutrients provided by fruits and vegetables, such as vitamins A, C, and E, polyphenols, and carotenoids, can increase and maintain cellular antioxidants to reduce oxidative stress. In fact, two meta-analyses have investigated the association between vitamins C and E and LC risk [98,99]. The results indicated that the risk of LC decreases by 7% with every 100 mg/day increase in vitamin C intake and that the risk of LC decreases by 14.2% with increasing dietary vitamin E intake [25]. However, the role of antioxidants in LC prevention remains controversial. Although epidemiological studies generally support a protective effect of antioxidant-rich foods, evidence from intervention trials has yielded conflicting results. Indeed, from the landmark Alpha-Tocopherol Beta-Carotene Cancer Prevention Study and the Beta-Carotene and Retinol Efficacy Trial, which demonstrated a higher risk of LC among smokers supplemented with β-carotene, to more recent data questioning the safety and efficacy of vitamin E supplementation, the role of antioxidant supplements in LC prevention continue to be highly debated [38,99].

These apparently contradictory findings underscore the dual nature of antioxidants in LC. On one hand, antioxidants can protect normal cells from ROS-induced DNA damage, thereby reducing mutagenesis and cancer initiation. On the other hand, once premalignant or malignant cells have emerged, the same antioxidant mechanisms may help cancer cells cope with oxidative stress, evade ROS-mediated cell death, and support tumor survival and progression. This dual role may explain why antioxidant-rich dietary patterns, particularly the MD, consistently demonstrate protective associations against LC, whereas supplementation with high doses of isolated antioxidants has not reproduced these benefits and may even increase cancer risk in susceptible populations [100].

Furthermore, isothiocyanates, indoles, flavonoids, and other phytochemicals may modulate multiple antitumor pathways through distinct mechanisms, including the inhibition of cancer cell invasion and tumor-induced angiogenesis, a process essential for tumor growth and progression, thereby potentially reducing LC risk. In addition, the bioactive compounds found in fruits and vegetables exhibit anti-inflammatory, antimicrobial, and antiviral properties that may further contribute to LC prevention. Consistent with these findings, a meta-analysis reported that each 20 mg/day increase in flavonoid intake was associated with a 10% lower risk of LC [29].

Cigarette smoking has also been associated with reduced circulating levels of provitamin A carotenoid concentrations, as suggested by the meta-analysis of Yu et al.: 18 studies out of 19 (comprising 10,261 lung cancer cases) have shown that a higher concentration of dietary β-carotene intake could reduce the risk of lung cancer [25].

The MD has been consistently associated with a reduced risk of LC, with the strongest protective effect observed among current smokers [24]. MD, in fact, is an anti-inflammatory diet, and the results of a meta-analysis revealed that the typical healthy foods of MD combined can fight against LC, probably more efficiently than any single food [83]. The protective effect is likely attributable to the antitumor properties of the bioactive compounds present in a healthy diet, including antioxidants, polyphenols, fiber, and minerals, which can inhibit cancer cell growth and progression [83]. Moreover, the interactions among these compounds may produce synergistic protective effects [26].

In contrast, a meta-analysis including 33 studies, of which six were prospective cohort studies, reported that each 120 g/day increase in red meat consumption was associated with a 35% higher risk of LC, while every 50 g/day increase in processed meat intake was associated with a 20% higher risk. However, epidemiological studies investigating the association between dietary cholesterol derived from meat and LC risk have yielded conflicting findings, and the available evidence remains inconclusive [24,27]. Therefore, the increased risk of LC associated with higher meat consumption does not appear to be attributable to its low-density lipoprotein (LDL) cholesterol content.

The gut microbiota may also influence the initiation and progression of LC. Through the lymphatic and blood circulation systems, a complex bidirectional axis connects the gut microbiota and lungs. According to previous studies, prebiotics and probiotics may have potential protective effects on the development of LC; however, the evidence is preclinical or qualitative, and well-designed clinical RCTs are still needed [101]. Consuming yogurt is linked to a 30% reduction in the risk of developing LC [102].

### 3.6. Ovarian Cancer

Ovarian cancer (OC) is the seventh most common cancer among women worldwide. Currently, no reliable screening methods are available for OC, and because early-stage disease is often asymptomatic, the majority of cases are diagnosed at an advanced stage [30,31]. Given the high mortality and substantial disease burden associated with OC, improving our understanding of its etiology and identifying effective preventive strategies are of paramount importance. Accordingly, preventive approaches, particularly those based on lifestyle and dietary modifications, are especially appealing because they are less costly and associated with fewer risks than medical interventions.

However, the relationship between diet and the risk of OC is not currently strongly evident [21,22]. Diet may influence the endogenous hormonal milieu, thereby contributing to the development of hormone-related cancers, including OC [21]. In particular, a pro-inflammatory dietary pattern, characterized by a high intake of foods rich in saturated fats and refined carbohydrates and a low intake of foods rich in PUFAs, flavonoids, vitamins, minerals, and other bioactive compounds, has been associated with an increased risk of OC [22,32].

Prolonged exposure to estrogens has been associated with an increased risk of hormone-related cancers. Accordingly, dietary patterns characterized by a high intake of fat and red meat have also been linked to a higher risk of these malignancies. Because estrogens and progesterone are steroid hormones synthesized from cholesterol, high-fat diets may provide an excess of substrates for estrogen biosynthesis, potentially promoting cell proliferation within the female reproductive tract. In addition, diets rich in animal-derived foods may increase exposure to xenoestrogens, exogenous compounds with potential carcinogenic properties [22]. Red meat and processed meat, in addition to being sources of saturated fat and iron, contribute to the formation of carcinogens, NOCs and heterocyclic amines [32].

Some evidence has also shown that increased whole milk consumption and lactose intake are associated with a greater risk of developing OC, particularly in African–American women [22,30,32]. Despite these findings, higher total calcium intake has been associated with a reduced overall risk of OC [32]. Calcium intake might downregulate circulating parathyroid hormone (PTH) levels. The reduction in PTH might reduce the hepatic and osteoblastic synthesis of insulin-like growth factor-1 (IGF-1), which exerts an effect by increasing cellular proliferation and inhibiting apoptosis. Reduced circulating IGF-1 levels may attenuate the mitogenic signaling involved in the pathogenesis of OC. In addition, calcium may protect against advanced-stage OC by mediating the pro-apoptotic effects of vitamin D [32].

However, these findings suggest that the potential adverse effects of the lactose and fat content of whole milk may outweigh the protective effects of calcium, thereby contributing to an increased risk of OC [30].

As mentioned above, a high-carbohydrate diet and fast food diet could increase OC risk. This may be explained by the fact that foods with a high glycemic index, such as those rich in refined sugars or white flour, induce a rapid rise in blood glucose levels, leading to an acute increase in insulin secretion. The resulting hyperglycemic and hyperinsulinemic state may promote glucose uptake by OC cells, thereby supporting their growth and proliferation to stimulate the growth of tumors, and acrylamide, which is a fast source of food, may cause the development of OC through its effects on sex hormones [22,31,32].

More generally, being overweight linked to this type of diet could increase the risk of OC due to the high concentration of leptin [22]. Leptin promotes the secretion of gonadotropin-releasing hormone (GnRH), which subsequently stimulates the release of luteinizing hormone (LH) and follicle-stimulating hormone (FSH). Elevated LH levels may induce premature ovulation, while increased circulating cholesterol, resulting from diets rich in saturated fat and excess energy intake, may enhance estrogen synthesis. Together, these hormonal alterations may impair normal ovarian re-epithelialization. Persistent ovarian stimulation may, in turn, promote abnormal cellular proliferation, thereby increasing the likelihood of malignant transformation [22].

These findings support the hypothesis that diets rich in anti-inflammatory foods may reduce the risk of OC. Consistent with this hypothesis, several studies have reported that higher consumption of fish, fruits, vegetables, particularly bulb vegetables, and whole-grain products is associated with a lower risk of OC [31].

In addition to providing ascorbic acid (vitamin C), vitamin E, and trace elements such as selenium, fruits and vegetables are important sources of B vitamins, including vitamins B6 and B12 as well as folate, which are essential for normal DNA replication and DNA repair [22].

Dried fruits are also important because of their high total phenolic content. Among these compounds, isoflavones, as well as certain flavones, flavanones, and flavanols, exhibit estrogenic or antiestrogenic activity, making them of particular interest for the modulation of the risk of hormone-related cancers, including OC [22,31].

An important role is played by dietary sources of n-3 PUFAs: these compounds may inhibit the promotion and progression of carcinogenesis through multiple mechanisms, including the modulation of estrogen metabolism, thereby reducing estrogen-induced cell proliferation [22,32].

### 3.7. Glioblastoma

Glioblastoma (GBM) is the most common and aggressive primary malignant brain tumor in adults, characterized by rapid growth, extensive invasiveness, and a poor prognosis. GBM are highly heterogeneous tumors and are the most frequently diagnosed primary brain tumors [102]. Exposure of the brain to oxidative stress is one of the most important risk factors for GBM. It can be mitigated by intake of antioxidants such as flavonoids, which are a class of polyphenolic compounds synthesized naturally by plants, and can be found both in normal diet and dietary supplements. Reduction in oxidative stress appears to be a common mechanism by which flavonoids protect glial cells [102]. Quercetin, catechins and proanthocyanidins all possess antioxidative activity, although proanthocyanidins and EGCG may also act as pro-oxidants. Furthermore, quercetin and some catechin derivatives, such as catechin and EGCG, protect glial cells and adjacent neurons against inflammation [102]. Preclinical studies suggest that omega-3 PUFAs, particularly DHA, may exert antitumor effects in glioblastoma by increasing ROS generation, promoting apoptosis, inhibiting tumor cell proliferation and migration, and enhancing the efficacy of chemotherapeutic agents such as lomustine and temozolomide. However, these findings are currently limited to experimental models, and clinical evidence supporting the use of omega-3 supplementation in patients with glioblastoma remains lacking [103,104].

To date, evidence linking adherence to the MD with glioblastoma risk is scarce and inconclusive. Although some observational studies suggest that higher consumption of fruits, vegetables, and antioxidant-rich foods may be associated with a lower risk of gliomas, no robust epidemiological evidence supports a protective effect of the MD specifically against glioblastoma [105]. Findings from a meta-analysis conducted by Shu et al. indicate that higher intakes of healthy dietary patterns, vegetables, and fruits are significantly associated with the lower risk of glioma [106]. A systematic review and dose–response meta-analysis suggested that the intake of tea, total vegetables, green vegetables, and orange vegetables may reduce the risk of glioma, whereas higher intakes of grains, processed meats, and processed fish may increase glioma risk. Therefore, the potential impact of dietary factors on glioma development should not be overlooked [107].

## 4. Nutritional Approaches in Cancer Therapies

Furthermore, accumulating evidence suggests that nutritional interventions play an important role in influencing cancer prognosis, improving patients’ quality of life, and enhancing the efficacy of antitumor therapies. In addition, adherence to a healthy diet, regular physical activity, and appropriate weight management are recognized as key components of supportive cancer care and have been associated with improved survival outcomes in patients with cancer.

**Figure 1:**
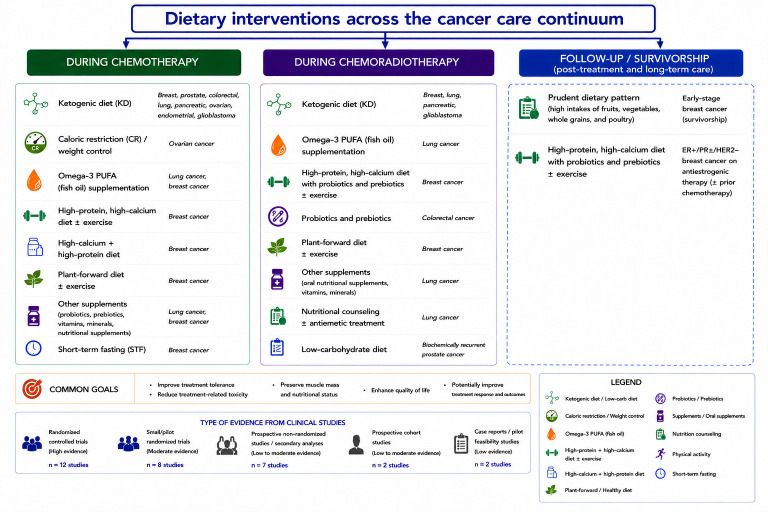
Overview of dietary interventions during chemotherapy, follow-up, and combined chemotherapy with radiotherapy. The figure summarizes the dietary interventions investigated in clinical studies during chemotherapy, combined chemotherapy with radiotherapy, and the follow-up/survivorship phase. Interventions include the ketogenic diet (KD), caloric restriction (CR)/weight control, omega-3 polyunsaturated fatty acid (PUFA) supplementation, a high-protein/high-calcium diet combined with exercise, plant-forward or healthy dietary patterns, and probiotics/prebiotics.

Therefore, nutritional intervention should be considered an integral component of a multimodal therapeutic approach aimed at reducing the risk of cancer recurrence, mortality, and the development of other chronic diseases [108]. The following section details the latest insights into nutritional approaches as adjunct strategies to conventional cancer treatments on the basis of studies of cell and animal models and clinical trials (Figure 1) (Table 2).

### 4.1. Impact of Dietary Approaches on Oxidative Stress

The Mediterranean diet (MD) is one of the most extensively investigated dietary patterns in relation to cancer prevention and supportive care. It is characterized by a high consumption of vegetables, fruits, legumes, whole grains, nuts, extra-virgin olive oil, and fish, together with a low intake of red and processed meat, refined carbohydrates, and ultra-processed foods [5,20]. This dietary pattern provides a wide variety of bioactive compounds, including polyphenols, carotenoids, vitamins C and E, selenium, dietary fiber, and omega-3 polyunsaturated fatty acids, which collectively contribute to maintaining cellular redox homeostasis [97]. The MD may counteract oxidative stress through several complementary pathways. First, dietary polyphenols, including hydroxytyrosol, oleuropein, resveratrol, quercetin, and flavonoids, directly scavenge reactive oxygen species (ROS) and reactive nitrogen species, thereby limiting oxidative damage to cellular macromolecules [54]. In addition, these compounds activate the nuclear factor erythroid 2-related factor 2 (Nrf2), a master regulator of the endogenous antioxidant response, leading to increased expression of cytoprotective enzymes such as superoxide dismutase (SOD), catalase, glutathione peroxidase, heme oxygenase-1 (HO-1), and NAD(P)H quinone oxidoreductase-1 (NQO1) [54]. Furthermore, several Mediterranean diet components have been shown to influence mitochondrial function, improving respiratory efficiency, limiting excessive mitochondrial ROS generation, and preserving cellular energy metabolism.

Plant-based dietary patterns have also attracted considerable interest as promising nutritional strategies for cancer prevention and supportive care. These dietary patterns emphasize the consumption of vegetables, fruits, legumes, whole grains, nuts, and seeds while limiting the intake of red and processed meats, refined carbohydrates, and ultra-processed foods. Consequently, they provide abundant dietary fiber, vitamins, minerals, and a broad spectrum of phytochemicals, including flavonoids, phenolic acids, carotenoids, lignans, and other polyphenols, many of which possess potent antioxidant and anti-inflammatory properties [1,96]. Collectively, these bioactive compounds contribute to maintaining cellular redox homeostasis by directly scavenging ROS and reactive nitrogen species (RNS), thereby reducing oxidative damage to DNA, lipids, and proteins. Furthermore, plant-derived phytochemicals modulate several redox-sensitive signaling pathways implicated in carcinogenesis [6,96]. Experimental studies have demonstrated their ability to activate the Nrf2, promoting the expression of endogenous antioxidant enzymes, including SOD, catalase, glutathione peroxidase (GPx), HO-1, and NQO1, while simultaneously suppressing the nuclear factor-κB (NF-κB) signaling pathway, thereby attenuating chronic inflammation and oxidative stress [3,6,96]. Overall, observational studies consistently associate greater adherence to healthy plant-based dietary patterns with lower cancer incidence and improved overall health; however, evidence from well-designed randomized controlled trials demonstrating a direct effect on cancer recurrence, progression, or survival remains limited [131].

Caloric restriction (CR) has been considered as one the most effective interventions for cancer prevention [132]. CR involves a sustained reduction in daily caloric intake without malnutrition, leading to long-term decreases in circulating glucose, insulin, and insulin-like growth factor-1 (IGF-1), together with improved metabolic flexibility and reduced chronic inflammation [132]. Nevertheless, available studies indicate a moderate degree of adherence within 1–4 months after dietary intervention. In this regard, alternate methods have been suggested as appropriate dietary regimens such as intermittent fasting (IF). IF involves short periods of marked energy restriction followed by periods of usual caloric intake. Several main regimens are described in the literature, differing in the length, frequency and circadian timing of fasting windows [133]. Short-term fasting (STF) refers to fasting regimens with a duration of 2–3 days and triggers an acute metabolic switch characterized by glycogen depletion, enhanced lipolysis, ketogenesis, and transient suppression of anabolic signaling [53]. In normal cells, fasting regimens have been shown to improve indices of mitochondrial efficiency and reduce mitochondrial ROS generation in specific organs. For example, fasting protocols can increase activities of complexes I and IV, normalize mitochondrial membrane potential and decrease superoxide production per unit of oxygen consumed [134]. In parallel, IF enhances cellular antioxidant defenses through a transient adaptive response [135]. STF initially induces a moderate increase in mitochondrial reactive oxygen species (ROS), which act as signaling molecules to activate redox-sensitive pathways, including PGC-1α, NRF2, and FOXO transcription factors. Following nuclear translocation, NRF2 binds to antioxidant response elements (AREs), promoting the transcription of genes encoding antioxidant enzymes, such as SOD1 and SOD2, as well as enzymes involved in glutathione synthesis and regeneration. Consequently, IF enhances mitochondrial antioxidant capacity, promotes a more reduced intracellular glutathione redox state, and increases the detoxification of superoxide, hydrogen peroxide, and lipid hydroperoxides. Collectively, these adaptive responses reduce oxidative damage to mitochondrial DNA, lipids, and proteins, ultimately lowering the overall oxidative burden over time [13613,^137^]. Studies in both in vitro and in vivo murine models have demonstrated that periodic fasting protects normal cells from oxidative stress while reducing cancer cell proliferation and enhancing tumor cell death during chemotherapy [9,137]. These effects are attributed to a phenomenon known as differential stress resistance (DSR), whereby IF induces a state of nutrient deprivation that suppresses tumor growth and proliferation while preserving adaptive stress responses in healthy cells. In contrast, cancer cells are unable to activate the same protective mechanisms, thereby selectively increasing their sensitivity to chemotherapy and enhancing its therapeutic efficacy [9]. These effects are largely mediated by the ability of fasting to lower circulating insulin and IGF-1 levels, leading to inhibition of the PI3K/AKT pathway. Because this pathway regulates cell growth, glucose metabolism, NADPH production, and cellular redox homeostasis, its suppression during fasting impairs the ability of cancer cells to cope with oxidative stress. Reduced PI3K/AKT signaling decreases glucose utilization and flux through the pentose phosphate pathway, resulting in lower NADPH production and impaired glutathione regeneration. Consequently, cancer cells become less capable of detoxifying reactive oxygen species (ROS), leading to increased oxidative burden and enhanced sensitivity to chemotherapy and radiotherapy [138,139,140]. In contrast, healthy cells respond to fasting by activating adaptive stress-resistance mechanisms, including FOXO-dependent antioxidant pathways, which preserve redox balance and protect against oxidative damage [3,140,141].

The ketogenic (KD), is a high fat, very low carbohydrate dietary intervention that induces a metabolic shift from glucose utilization to fatty acid oxidation and ketone body production. This type of diet leads to increased levels of ketone bodies in plasma while reducing the glucose concentration, creating a nutrient profile similar to that of long-term fasting [139].

In normal cells it was shown that KD is able to reduce oxidative stress due to its ability to directly scavenge free radicals and also to activate multiple protective antioxidant pathways [142]. Haces et al., has shown in vitro, both ketone bodies, beta-hydroxybutyrate (ßOHB) and acetoacetate (AcAc), are able to directly scavenge OH, while AcAc is able to scavenge hypochlorous acid (HOCl), ONOO^−^ and singlet oxygen (^1^O_2_). Prior studies have also shown that KD is able to activate the Nrf2/ARE system in both acute and chronic administration of ketone bodies and diet. Activation of the Nrf2 pathway protects cells from oxidative stress-induced cell death by upregulating expression of antioxidant proteins such as NQO1 and cytosolic (SOD1) and mitochondrial (SOD2) superoxide dismutase [143]. Furthermore, in a study conducted on rats, it has been demonstrated that ketones such as ßOHB and AcAc reduce glutamate-induced free radical formation by increasing the NAD^+^/NADH ratio and enhancing mitochondrial respiration in neocortical neurons. Thus, KD could be protective of healthy cells against oxidative damage [144].

Because many cancer cells exhibit increased glucose dependence and limited metabolic flexibility, a phenomenon often associated with the Warburg effect, the KD has been proposed as a complementary strategy to selectively target cancer cells [142]. Compared with normal cells, cancer cells have been demonstrated to be unable to utilize ketone bodies as an energy source and are believed to exhibit persistent mitochondrial oxidative stress, which is compensated for by increased glucose metabolism to generate reducing equivalents. Changes in mitochondrial structures and functions in cancer cells result in increased generation of ROS compared to healthy cells. It is advocated that cancer cells with higher glucose be used to aid in the breakdown of ROS. As a result, enhancing mitochondrial oxidative metabolism through techniques that limit glucose metabolism may cause oxidative stress in cancer cells. Thus, when KDs restrict glucose metabolism, lipid metabolism pushes cells to derive energy through mitochondrial metabolism, leading cancer cells to endure oxidative stress [145]. Tumor suppression by KD also appears to be attributable to its ability to reduce circulating insulin and IGF-1 levels, which are key activators of the PI3K pathway involved in cancer cell growth and survival, as well as to the dependence of malignant cells on glucose-derived metabolites, such as nucleotides and NADPH, to support their biosynthetic demands (Warburg effect) [127].

The antitumor effects of ketogenic diets may also be attributable to the intrinsic biological activity of free (non-esterified) long-chain fatty acids. (LCFAs) provided that they reach sufficiently high intracellular concentrations. Carbohydrate restriction may indeed limit the availability of glycerol-3-phosphate and insulin, which are required for the esterification of LCFAs into downstream lipid products [108].

Dietary modulation can also impact other metabolic cycles essential for cancer cell survival and antioxidant defence. Methionine restriction, another dietary approach, interferes with the S-adenosylmethionine (SAM) cycle, reducing the number of methylation reactions that support tumor growth. The SAM cycle is critical for methyl group donation during DNA methylation and histone modification, both of which regulate gene expression in cancer cells. It is also closely linked to antioxidant defence through the transsulfuration pathway, in which methionine-derived homocysteine is converted into cysteine, the late limiting precursor of GSH synthesis [146]. Furthermore, inhibition of methionine uptake disrupts these methylation processes, depriving cancer cells of the ability to sustain epigenetic modifications essential for their growth and survival [147].

Targeting cancer metabolism and antioxidant defence through dietary approaches such as glucose reduction and methionine restriction can impair key cellular pathways that support tumor growth, survival, and decrease the ability of cancer cells to neutralize ROS. These strategies not only disrupt the metabolic flexibility of cancer cells but also enhance the effectiveness of conventional therapies, offering a potential avenue for improved treatment outcomes [146,147].

### 4.2. Prostate Cancer

Currently therapeutic approaches for PCa, such as androgen deprivation therapy (ADT) and androgen receptor pathway inhibitors (ARPIs), have significantly improved oncological outcomes but are frequently associated with detrimental metabolic consequences, including increased adiposity, insulin resistance, and loss of muscle mass, which may negatively affect patient’s overall metabolic health and quality of life [58,148]. Tumor progression in PCa is largely driven by androgen receptor (AR) signaling, a pathway deeply interconnected with cellular metabolic reprogramming [58]. Preclinical studies have consistently shown that carbohydrate restriction and ketogenic dietary interventions can inhibit tumor growth, modulate critical oncogenic signaling pathways, including PI3K/AKT/mTOR, reduce systemic insulin, and improve survival outcomes in PCa models [58]. Furthermore, emerging evidence from xenograft murine models of metastatic prostate cancer suggests that ketogenic approaches may exert synergistic effects when combined with standard anticancer treatments, such as androgen deprivation therapy (ADT) and immunotherapy [58]. By lowering circulating glucose, insulin, and IGF-1 levels, the KD may counteract some of the endocrine and metabolic consequences of ADT and ARPI, while simultaneously exploiting cancer-specific vulnerabilities in glucose and lipid metabolism [149,150]. Early studies by Freedland et al. provided evidence that severe carbohydrate restriction may inhibit PCa growth in vivo. Using a xenograft model in SCID mice implanted with human LAPC-4 PCa cells, the authors demonstrated that animals fed a no-carbohydrate KD exhibited significantly slower tumor growth compared with mice receiving a Western diet, despite comparable or even higher caloric intake. This effect was partly explained by reductions in systemic insulin and modulation of the IGF pathway suppressing the PI3K/AKT/mTOR signaling cascade [151]. Emerging evidence has also focused on the potential application of KD to enhance the efficacy of immunotherapies (anti-PD-1/CTLA-4) in resistant PCa. Importantly, when combined with anti–PD-1 and anti–CTLA-4 antibodies, KD enhanced the antitumor response and significantly reduced tumor growth in preclinical models of otherwise resistant PCa [152]. These findings suggest that metabolic modulation through KD or ßOHB may represent a promising strategy to enhance the efficacy of immunotherapy in advanced or treatment-resistant PCa [152]. Although evidence from preclinical studies suggests promising antitumor effects of KD in PCa, as reflected by prolonged PSA doubling time, clinical data remain limited and definitive evidence regarding the long-term impact of KDs on oncological outcomes is still lacking. The CAPS2 trial was a randomized multicenter study evaluating a low-carbohydrate diet (LCD) in men with biochemically recurrent prostate cancer. After six months, the LCD intervention resulted in significant weight loss, improved insulin sensitivity, increased ketone levels, with a favorable trend toward longer PSA doubling time. Exploratory analyses showed a significantly prolonged PSA doubling time in the LCD group compared with controls, suggesting potential slowing of biochemical disease progression [153]. A secondary metabolomic analysis by Chi et al. demonstrated that a carbohydrate-restricted diet induced nutritional ketosis, characterized by increased circulating ketone bodies and alterations in lipid metabolism. Higher ketone levels were associated with a slower PSA doubling time, suggesting that enhanced ketogenesis may contribute to decelerating tumor progression in this patient population. These findings support the hypothesis that reducing glucose availability and promoting ketone metabolism could provide a metabolic disadvantage for prostate tumor cells [154]. Concerning the role of KD as preventive strategies for PCa, epidemiological evidence supporting KD is still scanty and current recommendations continue to emphasize healthy dietary patterns and weight management rather than specific KD interventions for primary prevention [48]. Consistently, recent studies showed how metabolic phenotype of PCa evolves throughout disease progression. If localized tumors largely rely on androgen-driven metabolic programs, advanced PCa exhibit greater metabolic plasticity, and it may partly explain the variable response to KD observed across preclinical and clinical studies and further supports the need for personalized nutritional strategies [155].

Moreover, a pilot prospective study investigated the effects of a periodic fasting-mimicking diet (FMD) on metabolic health parameters in patients with PCa. FMD cycles were safely implemented in a small cohort of PCa patients, with little or no observed toxicity and high overall adherence (83%). Analysis of metabolic variables showed an overall reduction in body weight, abdominal circumference, and blood pressure [156]. However, larger clinical trials focusing on metabolic risk factors, quality of life, and progression-free survival are needed to evaluate the potential effects of FMD in patients with prostate cancer.

### 4.3. Colorectal Cancer

Diet plays a fundamental role in CRC initiation, progression and prevention. In particular, a correct diet may have potential implications for the treatment of CRC, as it can influence a wide range of biological mechanisms, including cell signaling, apoptosis, and immune system regulation. In addition to its overlying influence on the gut microbiome. Interventions aimed at restoring eubiosis through probiotics, prebiotics, dietary modulation, or fecal microbiota transplantation (FMT) are under investigation as adjunct strategies to conventional therapies [75]. Understanding the complex interplay between the gut microbiota and CRC may provide new perspectives for personalized medicine and innovative treatment approaches in this common condition.

Diet plays a crucial role in modulating cellular responses to environmental stimuli, and a balanced nutritional regimen can enhance immune metabolism by increasing the cytotoxic activity of CD8^+^ tumor-infiltrating lymphocytes within the tumor microenvironment, thereby suggesting a potential role in improving cancer prognosis [156]. Moreover, weight loss is a common feature in patients with CRC and has long been associated with poor clinical outcomes. Consequently, nutritional status should be routinely assessed in patients undergoing nonsurgical oncologic treatment. Furthermore, dietary counseling is an essential component of supportive care for malnourished patients with gastrointestinal cancers receiving chemotherapy [157].

First, high consumption of vegetables, fruits and fibers is recommended. Vegetables and fruits are rich in natural compounds (especially flavonoids, carotenoids and phytoestrogens) that can selectively target tumor cells after disease onset and help tumor recurrence or metastasis. Moreover, one of the most significant properties of natural compounds is their ability to enhance tumor cell sensitivity to chemotherapeutic agents. It has been reported that quercetin and curcumin have a chemosensitive potential in malignant cell lines that could favor their use as adjuvant therapies in conventional treatment protocols [158].

Furthermore, lipid metabolism-related genes are currently of interest in precision nutrition studies. Lipids regulate a broad spectrum of cellular processes, including ATP production, the activation of key signaling pathways, and the maintenance of membrane organization and plasticity. Consequently, alterations in lipid metabolism can influence multiple stages of tumorigenesis, affecting both primary tumors and distant metastases [159].

Recent evidence has highlighted a role for low-density lipoprotein cholesterol (LDL-C) in regulating cell proliferation and differentiation, suggesting that hypercholesterolemia may contribute to cancer progression and metastatic dissemination. In addition, LDL-C has been implicated in the modulation of host immune responses. Indeed, adequate LDL-C levels are required for the optimal expression of Fc receptors and CD14, which are involved in monocyte-mediated phagocytosis, whereas high-fat diets have been associated with impaired macrophage antitumor activity.

Moreover, elevated LDL-C levels have been reported to suppress T-cell proliferation, further supporting a potential role for hypercholesterolemia in impairing antitumor immune responses [160]. Several studies suggest a potential association between cholesterol and metastatic progression. In particular, experimental evidence indicates that dietary cholesterol deprivation is associated with a reduced incidence of metastasis in CRC [160]. Therefore, reducing the sources of LDL-C from the diet could be a strategy to improve CRC prognosis.

On the other hand, recent evidence suggests that certain n-3 PUFAs may enhance the efficacy of chemotherapeutic agents when used as adjuvant therapy. in the management of CRC.

Many clinical and preclinical studies have shown that the dietary intake of n-3 PUFAs improves the efficacy of chemotherapeutic agents through the suppression of inflammation and the induction of cancer apoptosis. Moreover, supplementation with n-3 PUFAs appear to provide several clinical benefits for patients with colon cancer, including the alleviation of cancer-related cachexia, increased body weight, and preservation or enhancement of lean body mass, thereby contributing to an improved quality of life. Therefore, n-3 PUFAs may be used as adjuvants in CRC therapy [161,162].

In skeletal muscle, tumor-derived factors and chemotherapeutic agents activate intracellular signaling pathways that suppress protein synthesis and induce specific transcriptional programs, leading to autophagy and the degradation of myofibrillar proteins. For this reason, n-3 PUFAs are also needed, as they inhibit the transcriptional activation of muscle catabolism. Supplements with essential amino acids, such as leucine and arginine, are also recommended [163].

Probiotics and prebiotics also play important roles in anticancer therapy: they may also improve treatment tolerability by reducing the adverse effects associated with anticancer therapies, as demonstrated in several clinical trials. In particular, a recent systematic review and meta-analysis evaluated the efficacy of perioperative and postoperative probiotic supplementation as a therapeutic strategy for managing CRC treatment-related complications in patients undergoing surgery revealed reduced postoperative infectious complications and shorter hospital stays [164]. Probiotics have been shown to exert direct anticancer effects through multiple mechanisms, including the inactivation of carcinogens and mutagens, modulation of cell differentiation, and immunomodulatory activity [126].

Although a recent systematic review assessing 24 randomized controlled trials (RCTs) suggested possible benefits of microbiota modulation through supplementation with pre, pro, or synbiotics for reducing chemotherapy/radiotherapy side effects and potentially influencing treatment response in CRC patients, the findings are heterogeneous, and further large RCTs are needed [165].

Preclinical and early-clinical data suggest that FMT may help modulate gut dysbiosis in CRC through multiple mechanisms: restoring microbial diversity, reducing proinflammatory bacteria, improving chemotherapy-induced toxicity, and possibly enhancing immune responses in the tumor microenvironment [166]. However, human data are still very limited, most studies are in animal models, and large RCTs are needed to clarify whether FMT can play a role in CRC patients as an adjunct therapy [166].

The effects of a diet low in carbohydrates and macronutrients revealed that higher carbohydrate intake was associated with increased CRC mortality among patients with stage I and III cancer [167]. One possible explanation behind these results could be attributed to the fact that diets high in carbohydrates elevate blood glucose levels that induce insulin production. In turn, insulin leads to cell proliferation, inhibition of apoptosis and carcinogenesis through IGF1. Thus, KD, which is rich in fat specially from plant origin, has the potential to decrease the level of insulin circulating in the blood which could affect CRC progression [167]. In addition, KD seems to ease the side effects of chemotherapy, specifically nausea, fatigue, and prevent the loss of lean muscle [110]. Another prospective trial investigated the clinical response of patients with CRC at stage IV receiving chemotherapy combined with a KD administered for one year. Compared with patients receiving chemotherapy alone, those in the KD group showed a significantly higher overall response rate (60% vs. 21% in the control group) [109].

#### Antioxidant-Based Nutritional Interventions

Dietary antioxidants, including vitamins C, E, A, carotenoids, flavonoids, and other plant-derived bioactive compounds, have been investigated as potential nutritional strategies for CRC prevention. This interest derives from the role of oxidative stress in colorectal carcinogenesis and from the observation that diets rich in fruits, vegetables, whole grains, and fibre-containing foods are generally associated with lower CRC risk. However, when antioxidant compounds are examined individually, the clinical evidence appears heterogeneous and does not consistently support a clear protective effect. Meta-analytic data suggest that vitamin C may have a modest protective association, particularly when obtained through foods rather than supplements. The World Cancer Research Fund reported that foods containing vitamin C might decrease CRC risk, although the level of evidence was considered limited compared with stronger protective factors such as dietary fibre, wholegrains, dairy products, and calcium supplementation. Consistently, pooled analyses of prospective cohorts evaluating vitamins A, C, and E found inconsistent associations, indicating that antioxidant vitamin intake alone may not fully explain the protective effect observed for plant-rich dietary patterns [167]. Carotenoids have also been widely studied. Recent evidence from meta-analysis suggest that higher dietary carotenoid intake, as part of a whole-food dietary pattern, may be associated with lower cancer risk, whereas supplementation with isolated carotenoids remains controversial and may not reproduce the benefits of carotenoid-rich foods. [168]. Evidence from the SELECT trial further failed to support a preventive role for vitamin E, as supplementation did not affect colorectal adenoma occurrence compared with placebo (RR = 1.03; 0.96–1.10) [169]. Flavonoids, another major class of dietary antioxidants, have been evaluated in updated meta-analyses, and despite no association was observed between total flavonoid, flavanone, or flavan-3-ol intake and CRC risk, a high intake of flavonols was associated with a reduced risk of colon cancer (HR = 0.80, 95% CI: 0.68–0.94), but not rectal cancer (HR = 0.93, 95% CI: 0.74–1.18). Conversely, higher flavone intake was associated with a lower risk of rectal cancer (HR = 0.82, 95% CI: 0.70–0.97), whereas no significant association was observed with colon cancer risk HR = 0.88; 0.69–1.13) [170].

### 4.4. Breast Cancer

Patients with BC are frequently overweight or obese at the time of diagnosis, and obesity has been associated with increased BC-specific mortality. Moreover, even in the absence of weight gain, many women experience unfavorable changes in body composition, characterized by sarcopenia accompanied by increased adiposity. This condition represents a significant risk factor for the development of comorbidities, including cardiovascular disease and diabetes, thereby adversely affecting long-term survival [108,171,172].

A recent cohort study reported that each 5 kg increase in body weight following a BC diagnosis was associated with a 19% higher risk of cardiovascular mortality and a 13% higher risk of BC-specific mortality [173]. According to preclinical and clinical data, obesity may worsen the occurrence, severity, and mortality of BC [174]. Moreover, chemotherapy interferes with patients’ diet, negatively impacting the quality and intake of micronutrients and macronutrients and their nutritional status, with an increase in anthropometric measurements [171]. For this reason, among patients with BC, diet, regular physical activity, and appropriate weight management play a pivotal role in improving survival outcomes.

Numerous studies have reported improvements in multiple quality-of-life outcomes in patients with BC following healthy dietary patterns [124]. Adherence to a healthy dietary pattern characterized by a high intake of fruits, vegetables, whole grains, poultry, and fish, rather than a Western dietary pattern, may improve the overall prognosis and survival of women diagnosed with early-stage BC [115,117,171,172]. According to evidence from multiple studies, daily energy intake could be proposed for a nutritional plan for women with BC, which is distributed as follows: <30% fat/d (mainly monounsaturated fatty acids, MUFAs and PUFAs), 55% carbohydrates (mainly whole-food servings such as oats, brown rice, and fruits) and 1.2–1.5 g protein for a kg of body weight to avoid sarcopenia (which practically means a 25–30 g protein intake per meal for most patients) [113,175].

To counteract the Warburg effect, especially in patients receiving neoadjuvant chemotherapy, some studies have recommended reducing the percentage of carbohydrate intake from the common 55% to only 40% [113]. In every case, BC patients should be encouraged to consume 5–9 servings/d of fruits and vegetables.

Dietary fat intake should primarily derive from sources of omega-3 fatty acids, including fatty fish, walnuts, flaxseeds, chia seeds, and other nuts and seeds, as well as from sources of medium-chain triglycerides, such as fermented dairy products (e.g., yogurt, sour milk, and kefir) and coconut oil [113,171,175]. Omega-3 fatty acids should also be integrated because they are an adjuvant treatment for BC. These findings may help oncologists manage ongoing chemotherapy side effects by improving patients’ antioxidant status [176].

Vegetables and fruits consumed by patients with BC should be rich in β-carotene, vitamins A, E, and C, and flavonoids, as these bioactive compounds have been associated with improved BC outcomes and overall health [113].

Moreover, experimental studies indicate that many natural products present in foods can affect the progression of BC, such as soy (genistein and daidzein), pomegranate (ellagitannins), mangosteen (mangostin), citrus fruits (naringin), apple (2α-hydroxyursolic), grape, mango, cruciferous vegetables (isothiocyanates), ginger (gingerols and shogaols), garlic (organosulfur compounds), black cumin (thymoquinone), edible macrofungi (polysaccharides), and cereals. These foods should be included in the diets of women with breast cancer.

The anti-BC effects of these natural products are mediated through multiple mechanisms, including the inhibition of tumor cell proliferation, migration, metastasis, and angiogenesis, as well as the induction of apoptosis, cell cycle arrest, and enhanced sensitivity of tumor cells to radiotherapy and chemotherapy [177]. The MD represents a significant source of these bioactive compounds, so it can be chosen by BC patients [177].

Finally, Patients with BC should be encouraged to achieve and maintain a healthy body weight, corresponding to a body mass index (BMI) of 20.0–24.9, conserving lean mass and avoiding increased body fat mass. Therefore, after a nutritional status assessment, a conservative CR can be considered (500–1000 kcal/d) in personalized nutritional interventions if needed [113]. CR involves a sustained reduction in energy intake, leading to long-term improvements in insulin sensitivity, decreased circulating insulin and IGF-1 levels, reduced adiposity, and attenuation of chronic inflammation, all of which are implicated in BC progression [178]. Therefore, CR may influence BC biology by modifying the systemic metabolic environment that supports tumor development and recurrence. Caloric restriction (CR), typically involving a 20–40% reduction in caloric intake, has been shown to protect a wide range of organisms against oxidative stress and age-related decline Given its broad capacity to enhance cellular stress resistance, CR has been proposed as a potential therapeutic strategy for clinical application to protect patients from the toxic side effects of chemotherapy. However, this approach is not feasible for patients at risk of weight loss due to the underlying malignancy or chemotherapy [9].

Following the metabolic changes described above, STF further promotes adaptive stress responses through activation of AMPK and autophagy, accompanied by glycogen depletion, increased lipolysis, and ketone body production [9,111,114]. In long-term CR, humans have demonstrated reductions in metabolic and hormonal factors associated with cancer risk. Chronic CR is not considered a practical clinical strategy because it may exacerbate weight loss in patients with cancer [111]. However, a brief period of fasting may be feasible in patients, and in mice, a pilot study showed that an STF regimen slowed cancer growth at least as effectively as chronic CR without compromising body weight [111]. Furthermore, STF is considered a method to protect human cells from the harmful side effects of a variety of chemotherapy drugs [9,111]. Consistent with the phenomenon of differential stress resistance (DSR) described above, STF may increase the sensitivity of cancer cells to anticancer therapies while protecting normal cells from treatment-related toxicity. In a randomized cross-over pilot study, STF has been shown to induce extensive changes in gene expression and cellular metabolism that increase the resistance of normal cells to oxidative stress. These effects may be beneficial during cancer treatment with chemotherapy [114].

Another dietary approach identified as useful and adjuvant to chemotherapy in breast cancer is carbohydrate-restricted KD [108,118,127,179].

#### Antioxidant-Based Nutritional Interventions

The role of antioxidant supplementation in BC prognosis remains controversial, with evidence suggesting that its effects may vary according to the timing of administration, the specific nutrient involved, and the therapeutic context. A meta-analysis found no significant effects on the overall survival (HR = 0.92; 0.82–1.03) associated with antioxidant supplementation indicating no clear detrimental effect on long-term outcome [180]. Nevertheless, concerns have emerged regarding the use of antioxidants during active anticancer treatment, particularly considering the ROS mediated cytotoxic activity of several chemotherapeutic agents. The concurrent use of antioxidants—including vitamins A, C, and E, carotenoids, and coenzyme Q10 in patients receiving anthracycline- and taxane-based chemotherapy was associated with a 41% increase in the risk of disease recurrence (HR = 1.41; 0.98–2.04) and a 40% increase in the overall mortality (HR = 1.40; 0.90–2.18) [112]. These findings support the hypothesis that the antioxidant supplementation may interfere with ROS-mediated tumor cell killing, potentially reducing treatment efficacy. However, the interpretation of these results remains challenging. The evidence regarding individual vitamin supplementation is less consistent. In a cohort of 3405 women with invasive BC, those in the highest quartile of pre-diagnostic dietary vitamin C intake had a significantly lower risk of BC-specific mortality than those in the lowest quartile (HR = 0.75; 0.57–0.99). In contrast, vitamin C supplementation initiated after diagnosis was not associated with BC-specific survival, suggesting that the protective effects of vitamin C may be more pronounced when obtained through diet before cancer development [181]. A meta-analysis by Harris et al. reported that post-diagnostic vitamin C supplement use was associated with reduced mortality risk, while higher dietary vitamin C intake was associated with lower risks of both all-cause and BC-specific mortality [182]. Supporting a potential role in cancer prevention, a more recent meta-analysis of observational studies found that higher vitamin C intake was associated with a 14% reduction in BC incidence (RR = 0.86; 0.81–0.92) [183]. As a whole, the available evidence points toward a potential protective role of vitamin C in BC; however, the observed benefits appear to vary according to the timing of exposure, the source of intake, and the clinical context in which it is consumed.

### 4.5. Lung Cancer

Malnutrition, weight loss, and hypoalbuminemia are highly prevalent among patients with LC [184]. Accumulating evidence indicates that malnutrition adversely affects multiple clinical outcomes in patients with cancer, particularly those with LC, including treatment completion, survival, physical function, quality of life, and healthcare resource utilization. Emerging evidence provides some insight into which LC patients are at greater nutritional risk. Moreover, patients with LC receiving concurrent chemoradiotherapy appear to be at an increased risk of weight loss both during and after treatment [185,186]. Given the chronic nature of cancer and the sustained activation of the immune system, the diet should provide an adequate balance of all essential nutrients, including carbohydrates, proteins, fats, vitamins, and macro- and micronutrients. Otherwise, the body may begin to mobilize its own tissue reserves to meet these metabolic demands [187].

For this reason, it represents a fundamental dietary assessment and personalized nutritional support, with the aim of preventing or attenuating further weight loss. Such an approach should be an integral component of the rehabilitation care pathway for patients with LC [185]. In fact, a study by Tanaka et al. demonstrated that early individualized nutritional intervention initiated during chemotherapy may help maintain body weight compared with standard care and could be considered an adjuvant intervention to cancer therapy [124]. A well-balanced diet is crucial for supporting treatment in cancer patients even though it does not cure the disease [187].

As we have previously reported for other cancers, the nutritional value of anticancer therapy is conferred by omega 3 fatty acids [121,122,123,188]. Several chemotherapeutic agents (including anthracyclines, cisplatin, irinotecan, and alkylating agents) have been reported to show greater efficacy when fish oil supplementation (EPA and DHA) is added to the diet of patients with advanced non-small cell lung cancer or to cell culture media [121]. The distinct mechanisms of action of these antineoplastic agents suggest that fish oil may modulate the response to chemotherapy through multiple mechanisms. EPA and DHA may also exert antitumor effects by inhibiting angiogenesis and metastasis; moreover, they have presumable immune-modulating effects and, in particular, EPA has been shown to reduce the production of pro-inflammatory cytokines and mitigate cancer-associated tissue wasting [121,123]. Although the precise mechanisms remain to be fully elucidated, these findings suggest that fish oil may serve as an effective adjuvant to chemotherapy [121].

Finally, involuntary weight loss, a major contributor to morbidity and mortality in patients with advanced cancer, may be mitigated through nutritional interventions. Supplementation with fish oil-derived EPA has been shown to help prevent deterioration of body composition, as demonstrated in a randomized clinical trial [122].

Another dietary approach considered an adjuvant against LC could be the KD, as observed in other types of cancer [13,188,189,190]. A study has shown that consuming a KD administered during concurrent chemoradiotherapy appears to be clinically well tolerated in patients with locally advanced NSCLC and may exploit the altered oxidative metabolism of cancer cells to improve therapeutic outcomes [187,190].

Moreover, a KD could reduce the collateral effects of therapy: preclinical studies have shown that fasting and low-carbohydrate diets can reduce treatment-related side effects and enhance the efficacy of chemotherapy and radiotherapy, highlighting their potential for clinical application [191].

#### Antioxidant-Based Nutritional Interventions

Antioxidant supplementation has long been investigated as a potential strategy for reducing LC risk, particularly among smokers, owing to the central role of oxidative stress in lung carcinogenesis. However, the available evidence remains inconsistent and, in some cases, contradictory. While observational studies often support the protective role of antioxidant-rich diets, prospective studies and randomized clinical trials have generally failed to demonstrate a clear benefit of antioxidant supplementation. In particular, long-term supplementation with high doses ofβ-carotene, retinol, B vitamins, and vitamin E has not shown protective effects against LC incidence among current or former smokers [192,193]. Moreover, β-carotene supplementation was linked to a higher risk of LC in smokers, independent of the tar and nicotine content of the cigarettes consumed [194]. Conversely, the use of multivitamins and other dietary supplements, including ginseng, coenzyme Q10, and herbal preparations, has been associated with lower LC mortality in some cohorts of current smokers [195].

The heterogeneous findings observed across human studies may reflect the complex biological role of antioxidants in LC. Indeed, growing experimental evidence suggests that antioxidants can exert both protective and deleterious effects depending on the stage of tumor development and the molecular characteristics of the tumor. Several preclinical studies have demonstrated that antioxidant supplementation can enhance metastatic dissemination without significantly affecting primary tumor growth. In KRAS-driven LC mouse models, dietary antioxidants markedly increased lymph node and distant metastases, irrespective of p53 status [196]. Furthermore, LC cell lines derived from antioxidant-treated mice displayed a more invasive phenotype than those derived from untreated controls. Mechanistically, antioxidants may reduce oxidative stress to levels that favor the cancer cell survival and metastatic competence, thereby facilitating tumor progression.

Collectively, these findings highlight the dual and context-dependent nature of antioxidant supplementation in LC prevention. While adequate antioxidant intake through a balanced diet rich in fruits and vegetables appears to contribute to reducing LC risk, the use of high-dose antioxidant supplements cannot currently be recommended as a universal preventive strategy.

### 4.6. Ovarian Cancer

Research in the literature has revealed mainly evidence related to the use of dietary approaches as adjuvants to chemotherapy in patients with OC. As we have already shown, the dependence of cancer cells on aerobic glycolysis renders them vulnerable to glucose and insulin deprivation. Accordingly, carbohydrate-restricted diets, such as the KD, may represent a non-pharmacological strategy to disrupt cancer cell growth and proliferation [119,197]. In a study by Cohen et al., among women with ovarian or endometrial cancer, a 12-week KD resulted in selective reductions in total and visceral adiposity, while preserving lean body mass and decreasing circulating levels of cancer-related growth factors [197]. Another randomized controlled trial confirmed this finding and showed how a KD may improve physical function, enhance energy levels, and reduce specific food cravings [120].

Other studies have proposed CR as an adjuvant strategy. By restricting caloric intake, a metabolic environment is created that limits the availability of nutrients required to fuel cancer cell growth. In contrast, normal cells are better able to utilize alternative energy sources, such as lipids and proteins, to meet their metabolic demands. In addition, when nutrient availability is limited, the body activates endogenous metabolic pathways to mobilize internal energy and nutrient reserves; in this context, tumor tissue may also be catabolized as a source of organic substrates [119]. However, women with this cancer tend to have a higher incidence of malnourishment, and cancer treatments such as surgery and chemotherapy may further compromise nutritional status by causing adverse effects, including short bowel syndrome, diarrhea, malabsorption, and fatigue. Thus, malnutrition in OC patients is frequent and has a multifactorial origin; for this reason, CR is not always applicable [198].

A diet higher in vegetables and fruit is always recommended, as it is associated with longer survival. Higher total vegetable intake was associated with improved OC survival. A prospective cohort study from the Women’s Health Initiative, including 341 cases and matched controls, suggested that higher consumption of vegetables, particularly green and orange-yellow vegetables, was associated with a 39% longer survival [199].

Other strategies identified in the literature are chewing gum and consuming coffee during the postoperative period. They have been shown to be safe, well tolerated, and effective strategies for accelerating intestinal recovery in patients with gynecologic cancers, including OC [198].

#### Antioxidant-Based Nutritional Interventions

Among antioxidant compounds, vitamin C has attracted considerable interest in OC research; however, current epidemiological evidence does not support a significant association between vitamin C consumption and OC risk. A meta-analysis including 16 studies found no significant effect of dietary vitamin C intake on OC incidence (RR = 0.95; 95% CI: 0.81–1.11) [200]. In contrast, growing interest has been directed toward the use of intravenous vitamin C as an adjunct to conventional therapies. High-dose intravenous vitamin C administered in combination with carboplatin and paclitaxel may enhance the sensitivity of tumor cells to standard chemotherapy in patients with stage III–IV OC [201]. Regarding the putative role of vitamin C as a preventing agent, some case–control studies have reported a protective association, showing that higher dietary vitamin C intake was linked to a substantially lower risk of OC. Patients consuming more than 363 mg/day of vitamin C exhibited a 45% lower risk of OC (OR = 0.45) [202]. In addition, a prospective cohort study conducted between 2015 and 2020 found that higher pre-diagnostic dietary vitamin C intake was associated with improved survival among patients with OC after a median follow-up of 37.19 months (HR = 0.43) [203]. However, other investigations have failed to confirm these beneficial associations. Several case–control and cohort studies found no significant relationship between vitamin C intake and ovarian cancer risk [200,204,205].

Consistently a pooled analysis of 10 prospective cohort studies involving 501.857 women and up to 16 years of follow-up found no association between vitamin C intake, whether from dietary or supplemental sources, and ovarian cancer risk [206]. Furthermore, a large prospective study from the California Teachers Study cohort reported that women with the highest vitamin C intake (>665 mg/day) had a significantly increased risk of OC compared with those consuming ≤75 mg/day [207].

### 4.7. Glioblastoma

Malignant brain tumors remain associated with a poor prognosis despite aggressive multimodal treatment, including surgical resection, chemotherapy, and radiotherapy. The median overall survival of patients with newly diagnosed glioblastoma is approximately 18 months [208,209]. The poor prognosis of patients with brain tumors underscores the need for the development of novel therapeutic strategies, particularly those that enhance the efficacy of existing treatments and/or inhibit tumor growth One emerging therapeutic strategy is to target metabolic dysregulation, which results in an increased need for glucose and possibly for glutamine in tumor cells [209,210]. This observation suggests that reducing carbohydrate availability through a low-carbohydrate KD may inhibit tumor growth [211].

Currently, few clinical trials are evaluating the effects of a KD on GBM, but these studies have demonstrated that a KD is safe and feasible, although it may not elicit significant benefits as a monotherapy in these patients [128,129,208]. Regarding safety, The ERGO trial assessed the feasibility of KD in 20 patients with recurrent GBM. Participants followed a low-carbohydrate KD enriched with plant oils. Although three patients (15%) discontinued the diet because of poor tolerability, no serious adverse events attributable to the dietary intervention were observed, indicating that KD was generally feasible and safe in this patient population [212].

Although initially proposed to act primarily by reducing glucose availability, ketogenic therapy for central nervous system (CNS) tumors, including gliomas, is now recognized to exert antitumor effects through a broad range of biological mechanisms. These include the attenuation of inflammation and oxidative stress, enhancement of antitumor immunity, modulation of gene expression, and increased sensitivity of tumor cells to standard-of-care and adjuvant therapies. Accumulating evidence suggests that ketone bodies may play a central role in mediating the therapeutic effects of ketogenic therapy in CNS tumors. Indeed, some of these mechanisms appear to be directly driven by ketone signaling, indicating that elevated circulating ketone levels may represent a key component of the therapeutic response [208,209]. These metabolic changes improve survival in animal models of malignant glioma and may potentiate the antitumor effects of chemotherapy and radiation treatment [213].

The first clinical application of KD in patients with malignant brain tumors was reported by [214]. The study included two girls with advanced, unresectable brain tumors (stage IV anaplastic astrocytoma and stage III cerebellar astrocytoma) who had previously received extensive radiotherapy and chemotherapy. The dietary intervention was designed to induce ketosis, thereby reducing glucose availability to tumor cells while preserving the patients’ nutritional status. Both patients exhibited a marked reduction in tumor glucose uptake and achieved prolonged disease control, supporting the potential therapeutic role of KD in malignant brain tumors [213,215].

Moreover, in a prospective pilot study involving patients with glioblastoma multiforme undergoing standard treatment, Champ et al. demonstrated that a ketogenic diet was feasible and well tolerated, inducing metabolic changes characterized by increased ketone levels and reduced glucose availability, supporting its potential as an adjuvant metabolic therapy [216]. CR, in addition to KD, can provide further advantages by activating sirtuin (*SIRT1*) genes that have been shown to inhibit tumor proliferation. For example, the *SIRT1* and *NRF-2* genes are both activated by CR. *SIRT1* has been reported to suppress both neurodegenerative processes and tumor development. *NRF-2* enhances more than 200 additional antitumorigenic genes. Experimental CR regimens reduce the circulating levels of IGF-1, vascular endothelial growth factor (VEGF), and cytokines, thereby attenuating growth factor signaling, reducing vascular abnormalities, and suppressing inflammation [217].

In addition, other dietary approaches have been shown to induce anticancer metabolic changes. Sulforphane is a sulfur-containing molecule found naturally in cruciferous vegetables, such as broccoli, Brussel sprouts, and cabbage, and has been shown to have anticancer and antimicrobial properties. Dietary antioxidants, such as curcumin (from turmeric) and resveratrol (from grapes and red wine), have also shown powerful anticancer properties, which may be helpful in the treatment of GBM [217,218]. Supplementation (vitamins, minerals and concentrated polyphenols) is also important as an adjuvant to both metabolic and chemotherapy treatments for CNS tumors [218]. In particular, the flavonoid kaempferol induced apoptosis in glioma cell lines by elevating intracellular oxidative stress. Heightened oxidative stress was characterized by an increased generation of ROS accompanied by a decrease in oxidant-scavenging agents such as SOD1 and thioredoxin (TRX-1). Furthermore, kaempferol inhibited glioma cell migration in a ROS-dependent manner and potentiated the toxic effect of chemotherapeutic agent doxorubicin by amplifying ROS toxicity [219]. Quercetin and EGCG, proanthocyanidins from the grape seed extract and cranberry juice, induce the arrest of GBM cell cycle and cell death by both apoptotic and non-apoptotic mechanisms. In addition, quercetin and EGCG inhibit the pro-oncogene MAPK signalling pathway and EGCG also intensifies the effects of both ionizing radiation and chemotherapeutic drugs on GBM cells [102]. However, most of the effects of flavonoids have been observed only in vitro and in vivo, while clinical studies on humans are lacking. Due to limited ability of the flavonoids to access the brain, their normal dietary intake is likely insufficient to produce significant anti-cancer effects in this organ, and supplementation is needed to increase brain concentration of flavonoids. It was shown that Ɣ-linolenic acid (GLA), DHA and EPA are able to increase the cytotoxic effect of radiotherapy in malignant rat astrocytoma cells [220]. Although GLA induces oxidative stress and apoptosis in rat glioma cells, it does not affect the viability of normal rat astrocytes, an effect that was associated with a differential expression in antioxidant enzymes [221]. In a small clinical study, patients undergoing surgical treatment for gliomas received GLA following surgery. Computed tomography imaging demonstrated a significant increase in tumor necrosis after GLA administration. Although limited by the small sample size, these preliminary findings suggest that GLA may have therapeutic potential in glioma management, with no significant treatment-related adverse effects observed [222].

#### Antioxidant-Based Nutritional Interventions

Oxidative stress is increasingly recognized as a central driver of GBM initiation, progression, and therapeutic resistance, exerting profound effects on tumor cell proliferation, survival, invasion, and adaptation to hostile microenvironmental [223]. ROS contribute not only to gliomagenesis but also to the remodeling of the tumor microenvironment, influencing immune cell function and promoting tumor-supportive signaling networks [224]. The biological effects of ROS are highly context-dependent, and GBM cell lines exhibit a pronounced capacity to adapt to chronic oxidative stress, activating antioxidant defense mechanisms that enable them to tolerate elevated ROS levels and, paradoxically, become more resistant to conventional therapies [225]. Although chemotherapy and radiotherapy exert part of their antitumor activity through ROS generation, the resulting oxidative pressure may also select for highly resilient glioma stem cells. The survival and expansion of these therapy-resistant cell populations are thought to contribute significantly to tumor recurrence and disease progression. Consequently, modulation of redox homeostasis and targeting oxidative stress-related pathways have emerged as promising therapeutic strategies aimed at overcoming resistance mechanisms and improving clinical outcomes in patients with GBM [225,226]. Nowacka et al. summarized evidence from multiple experimental models, including human glioblastoma cell lines, glioblastoma stem-like cells, three-dimensional spheroid models, and murine xenograft models, highlighting the role of oxidative stress and antioxidant pathways in GBM progression and therapy response [226]. Although oxidative stress is widely recognized as a hallmark of glioma, the available literature is characterized by substantial heterogeneity, making it difficult to draw definitive conclusions. However, the most comprehensive systematic review and meta-analysis currently available suggests that not all antioxidants exert equivalent effects. While a high dietary intake of vitamin C was associated with a significantly lower risk of glioma, no comparable protective association was observed for vitamins A and E. The relatively low incidence of gliomas, together with the limited availability of clinical data, makes it challenging to investigate the associations between dietary antioxidant vitamin intake and glioma subtypes, including glioblastoma.

A meta-analysis of observational studies by Lv et al., indicates how high intake of dietary vitamin A was significantly correlated to a reduced risk of glioma (RR = 0.80, 95% CI = 0.6–0.98, *p* = 0.014) [227]. In contrast, Ni et al. performed an updated systematic review and meta-analysis of observational studies, showing no significant association between dietary vitamin A intake and the risk of glioma (RR = 0.78; 95%CI: 0.57–1.05; *p* = 0.103) [228]. Vitamin C was one of the most extensively investigated antioxidant vitamins in relation to the glioma risk, although the available epidemiological evidence remains inconsistent. While several studies have reported no significant association between vitamin C intake and glioma incidence, an earlier meta-analysis found that higher vitamin C consumption was associated with a significantly reduced risk of glioma, particularly among US populations [229]. However, not all studies have confirmed this relationship, as three large prospective cohort studies conducted in the United States reported essentially null associations between vitamin C intake and glioma development [230]. To date, the American Brain Tumor Association firmly recommends that patients focus on eating real food rather than isolating individual nutrients or vitamin supplements [231].

## 5. Conclusions and Future Perspectives

Among the dietary patterns investigated, MD has emerged as one of the most promising approaches for reducing cancer risk and mortality. It is characterized by a high intake of legumes, cereals, fruits, nuts, vegetables, extra-virgin olive oil, and PUFAs, together with moderate consumption of red wine and limited intake of red meat, poultry, and dairy products.

Current evidence supports the antioxidant, anti-inflammatory, and immunomodulatory effects of spices, which may contribute to the prevention and treatment of several cancers, including lung, colorectal, and breast cancer. Several spices are assumed to be potential sources for the prevention and treatment of cancers because they contain several important bioactive compounds. The principal mechanisms of action include the induction of apoptosis, inhibition of tumor cell proliferation, migration, and invasion, and enhanced sensitivity of tumor cells to radiotherapy and chemotherapy [232]. It would also be ideal to expand this type of study on humans to improve the bioavailability of these compounds, which could easily become part of the diet of patients undergoing chemotherapy, enriching the flavor, taste and color of food in any type of diet. Various studies on animals have shown that combination treatment is a potential therapeutic strategy for cancer and that spices are promising sources of adjuvant cancer therapy. The most popular are studies on curcumin. Ashrafizadeh et al. demonstrated that curcumin is able to enhance the antitumor activity of paclitaxel against different cancers in mice and that curcumin administration reduces the adverse effects of this chemotherapy [233]. Additionally, Kumar et al. demonstrated the anticancer potential of curcumin in animal models when it was given in combination with chemotherapeutics such as cyclophosphamide, doxorubicin and mitomycin [234]. This topic has also been addressed both in vitro and in vivo recently by Tan et al. [116]. There are many spices that we can still consider adjuvants to anticancer therapy, especially as aids to reduce its adverse effects. Future research should focus on the identification and characterization of the bioactive anticancer compounds present in spices, as well as on elucidating their mechanisms of action and evaluating their efficacy in human studies.

The KD is among the dietary approaches investigated as an adjunct to antitumor therapy. Accumulating evidence suggests that low-carbohydrate, high-fat diets may delay tumor progression and enhance the effectiveness of conventional cancer treatments [235]. Although preclinical evidence from both in vitro and in vivo studies suggests that KD may enhance the efficacy of existing cancer therapies, clinical evidence remains insufficient to establish its therapeutic effectiveness because of the limited number of well-designed trials and the lack of standardized KD protocols.

Rigorous dietary monitoring and objective assessment of ketosis are essential for accurately evaluating the therapeutic efficacy of KD [236]. The majority of clinical studies on the antitumor effects of KDs have focused on specific types of cancer, including brain, prostate, breast, ovarian, and lung cancer. Nevertheless, there are a few studies that have addressed such effects on CRC and other cancers, and we can draw information and hope only from preclinical studies performed on mice or xenografts to determine their efficacy. One of the earliest studies was conducted by Nakamura et al., who investigated the antitumor and anticachectic effects of KD in mice bearing colorectal tumors. At the end of the intervention, mice receiving KD exhibited significantly higher blood β-hydroxybutyrate (βHB) concentrations, which were inversely correlated with tumor weight Furthermore, the increase in the plasma IL-6 concentration was inhibited by the increased blood concentration of βHB, and in the treated mice, the body weight did not decrease compared with that in the control group [237]. These findings suggest that KD may slow cancer progression while attenuating the associated systemic inflammatory response, without compromising body weight or skeletal muscle mass, thereby potentially reducing the risk of cancer cachexia. In a subsequent study, Hao et al. evaluated the effects of KD in a murine model of colorectal cancer established by subcutaneous inoculation of HCT116 colon cancer cells into 36 male mice [238]. The animals were then randomly assigned to different dietary intervention groups, and the treated groups were fed a KD rich in omega-3 fatty acids and medium-chain triglycerides. The tumor growth in the treated group was significantly delayed. Therefore, these results suggest that a KD may also provide a promising strategy for antitumor therapy in the future for CRC. Preclinical data have demonstrated that the antitumor effects of KD are multifaceted and that modulation of energy metabolism may suppress cancer cell proliferation and enhance tumor sensitivity to anticancer therapies.

In addition, CR and fasting, as adjuvants to conventional treatments can improve the patient’s survival and the response to therapy. Across studies, interpretation of the findings is constrained by brief intervention periods, poor adherence to the prescribed diets, and substantial interindividual variation in metabolic responses [116]. Currently, there are no large RCTs on the clinical efficacy of these nutritional strategies in humans. To establish the clinical utility of these approaches, particularly within the context of personalized medicine, larger prospective studies with adequate power to evaluate overall survival and disease-free survival are required. In particular, these studies will have to focus on the impact of a KD, CR and fasting for extended periods and identify patient subtypes that are more suitable for these strategies. Thus, larger RCTs focused on a deeper evaluation of diet effects as adjuvants to chemotherapy and radiotherapy and on tumor invasive growth and metastasis might allow the combination of personalized nutritional approaches and conventional anticancer treatments in the very near future. Recent advances in precision nutrition are transforming the role of dietary interventions in cancer prevention and treatment. Rather than adopting a “one-size-fits-all” approach, precision nutrition aims to tailor nutritional strategies according to an individual’s metabolic phenotype, genetic background, lifestyle, nutritional status, anti-cancer treatments, comorbidities and gut microbiome composition. This personalized approach recognizes that interindividual variability in nutrient metabolism and host–microbiome interactions may substantially influence cancer susceptibility, disease progression, and response to therapy [239]. Metabolic phenotyping has emerged as a valuable tool for identifying patients who may benefit from specific dietary interventions. Alterations in glucose, lipid, and amino acid metabolism, together with differences in insulin sensitivity, inflammatory status, and body composition, can influence tumor biology and determine the effectiveness of nutritional strategies such as CR, KD, or MD patterns [131,240,241]. Integrating metabolomic profiling with clinical and molecular characteristics may therefore facilitate the development of personalized nutritional interventions aimed at targeting metabolic vulnerabilities of individual tumors while preserving the nutritional status of the patient [131].

**Figure 2:**
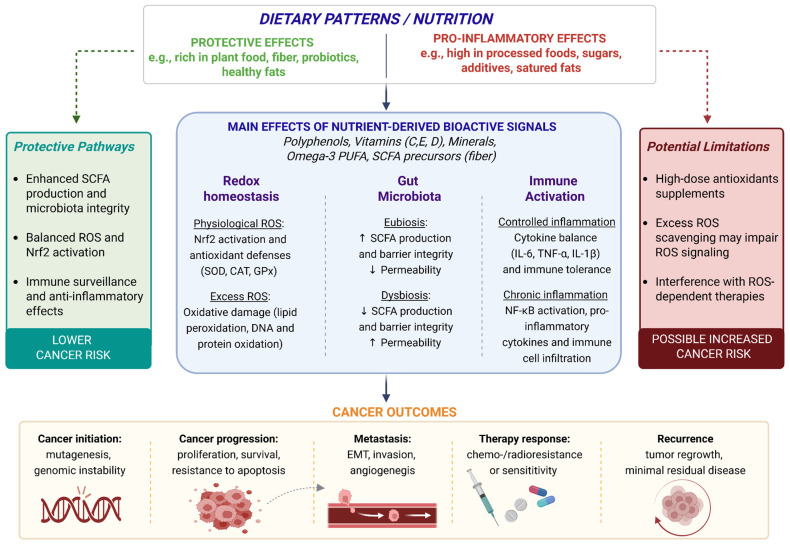
Overview of the molecular pathways through which dietary patterns influence cancer aetiology and progression. Nutrient-derived bioactive compounds modulate the tumor microenvironment through a complex interplay among redox homeostasis, gut microbiota composition, and immune-inflammatory signalling. While protective dietary patterns may support these processes and contribute to cancer prevention when consumed as part of a balanced diet, the indiscriminate use of high-dose antioxidant supplements may disrupt physiological reactive oxygen species (ROS) signalling, interfere with ROS-dependent anticancer therapies, and potentially compromise clinical outcomes in specific settings. Created in BioRender. Morosini, C. (2026). https://BioRender.com/9r2b3dx. accessed on 1 July 2026.

The gut microbiome has also gained considerable attention as a key mediator of diet–host interactions in oncology. Dietary components shape the composition and metabolic activity of intestinal microorganisms, which in turn produce bioactive metabolites capable of modulating inflammation, immune function, epithelial barrier integrity, and cancer-related signaling pathways [72,73,74,75,76,77,78,79,80,81]. Moreover, accumulating evidence indicates that gut microbiota composition influences the efficacy and toxicity of chemotherapy, radiotherapy, suggesting that microbiome-targeted nutritional interventions may enhance therapeutic outcomes [242,243]. In addition to tumor biology and individual metabolic characteristics, nutritional interventions should also be tailored according to the anticancer treatment regimen, baseline nutritional status, and the presence of comorbidities [69]. Different therapeutic modalities, including chemotherapy, radiotherapy, immunotherapy, and targeted therapies, are associated with distinct metabolic demands and treatment-related toxicities that may influence nutritional requirements and the safety of specific dietary interventions [69]. Likewise, nutritional strategies should be adapted to the patient’s nutritional status, as individuals with malnutrition, sarcopenia, or cancer cachexia may not tolerate restrictive dietary approaches such as fasting or caloric restriction [237]. Furthermore, common comorbidities, including diabetes mellitus, obesity, cardiovascular disease, chronic kidney disease, and hepatic dysfunction, may substantially modify metabolic responses and limit the applicability of certain nutritional interventions [69]. Consistent with the principles of precision nutrition, a recent systematic review by McLuskie et al. concluded that no single nutritional intervention can currently be recommended for all patients with incurable solid cancers [244]. Instead, the available evidence supports individualized nutritional strategies tailored to the patient’s clinical condition, nutritional status, treatment-related needs, comorbidity profile, and metabolic characteristics. Integrating these factors with microbiome profiling into precision nutrition strategies may enable the development of personalized interventions that improve treatment tolerance, enhance therapeutic response and clinical outcomes, support cancer prevention, and minimize the risk of adverse effects (Figure 2).

The history of antioxidant dietary supplementation in cancer prevention has been marked by far more controversies than successes. Although antioxidant supplementation was hypothesized to protect against carcinogenesis by reducing oxidative damage, many intervention studies have failed to confirm such benefits. Despite the strong biological rationale supporting their use, numerous intervention studies have failed to demonstrate consistent clinical benefits. Recently, data from SELECT study further strengthened the growing body of evidence from large-scale randomized trials indicating that high-dose antioxidant supplementation may paradoxically promote carcinogenesis rather than prevent it [38,52,169,245]. Although nutritional strategies play a pivotal role in cancer prevention and may support therapeutic outcomes, the use of dietary supplements remains a complex and often controversial issue. As highlighted in a *Nature* editorial discussing the current evidence on vitamin supplementation, the clinical effects of nutritional interventions are highly context-dependent and are influenced by baseline nutritional status, dosage, individual genetic and metabolic variability, and the methodological quality of intervention studies [246]. Moreover, the relationship between nutrient intake and health outcomes is unlikely to be linear, as both deficiency and excessive intake may adversely affect physiological homeostasis. In this context, excessive antioxidant supplementation may disrupt redox signaling pathways and potentially reduce the efficacy of anticancer therapies that rely on oxidative stress.

Boosting ROS scavenging may attenuate oxidative stress-induced apoptosis and interfere with the cytotoxic effects of chemotherapy and radiotherapy. Indeed, several anticancer treatments such as anthracyclines, cisplatin, gold (I)-phosphine compounds exploit the relatively high basal oxidative burden of malignant cells by further increasing ROS production beyond a tolerable threshold, thereby inducing oxidative DNA damage, mitochondrial dysfunction, lipid peroxidation, apoptosis, and other forms of regulated cell death. While reinforcing antioxidant defenses during treatment may protect healthy tissues from oxidative damage, it may also reduce the susceptibility of malignant cells to ROS-mediated cytotoxicity attenuating the therapeutic efficacy. In vitro, pretreatment with NAC has been shown to reduce cisplatin-induced oxidative damage, DNA fragmentation, and apoptosis, demonstrating that the scavenging ROS can attenuate cellular responses contributing to platinum-induced cytotoxicity [247]. Again, mouse models of BRAF- or KRAS-driven lung cancer, reported how dietary supplementation with NAC or vitamin E reduce ROS levels and DNA damage, inhibit p53 activation, accelerating tumor growth, and shortened survival as well as the administration of NAC increases lymph node metastases in malignant melanoma mouse model [49,248]. Although, natural antioxidants may protect healthy tissues under some conditions without necessarily reducing antitumor efficacy, particularly when their administration is temporally separated from chemotherapy, caution is warranted regarding the indiscriminate use of high-dose antioxidant supplements during chemotherapy or radiotherapy in the absence of a documented nutritional deficiency and careful clinical evaluation. Indeed, recent prospective studies indicate that use of antioxidant supplements during chemotherapy, as well as iron and vitamin B12, may increase the risk of breast cancer recurrence and mortality [112].

**Figure 3:**
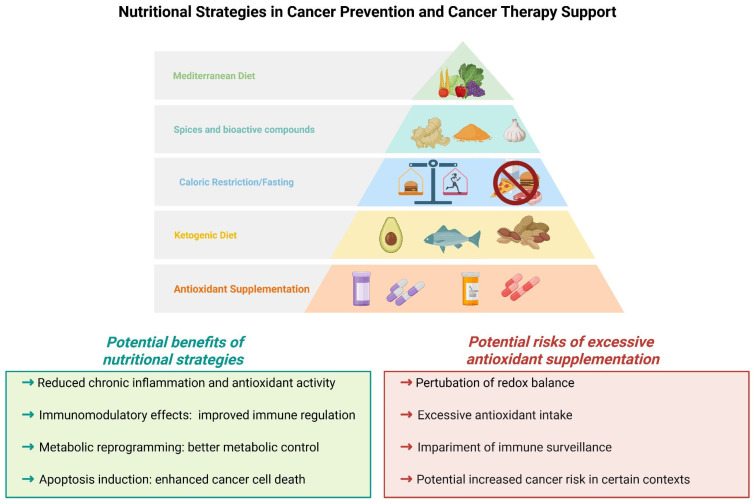
Nutritional strategies as potential adjuvant approaches in cancer prevention and therapy. The figure summarizes the main nutritional interventions investigated for cancer prevention and supportive cancer care, organized according to the current strength of scientific evidence. The Mediterranean diet represents the dietary pattern supported by the most consistent evidence, whereas spices and food-derived bioactive compounds, caloric restriction/fasting, and ketogenic diets show promising but still limited clinical validation. These strategies may exert anticancer effects through multiple biological mechanisms, including anti-inflammatory and antioxidant activities, immunomodulation, metabolic reprogramming, and apoptosis induction. However, the available evidence remains heterogeneous, with many findings derived from preclinical studies. Notably, the potential risks associated with nutritional interventions are primarily related to excessive or unbalanced dietary practices, rather than to the consumption of whole foods as part of a balanced diet. In particular, high-dose antioxidant supplementation may disrupt physiological redox signaling, impair immune surveillance, interfere with the efficacy of anticancer therapies, and, in specific contexts, potentially increase cancer risk. Likewise, restrictive or unbalanced dietary regimens (i.e., *monodiets*) may lead to nutritional deficiencies, metabolic alterations, and reduced treatment tolerance, especially in vulnerable patients with cancer. Overall, the clinical implementation of nutritional interventions should rely on a personalized, evidence-based approach and be supported by further randomized controlled trials. Created in BioRender. Morosini, C. (2026) https://BioRender.com/rstv0vu, accessed on 1 July 2026.

To date, large trials suggest that supplementation should not be considered a universal preventive or therapeutic strategy but rather a personalized intervention, guided by documented nutritional deficiencies, patient characteristics, and robust clinical evidence (Figure 3).

Overall, nutritional interventions show promising biological mechanisms that may influence cancer development, progression, and treatment response. However, current clinical evidence remains limited by small sample sizes, heterogeneous study populations, variability in dietary protocols, differences in treatment regimens, and the lack of standardized outcome measures, precluding definitive recommendations for routine clinical practice [69,131] (Table 3). Future research should focus on the development of standardized nutritional protocols and well-designed, adequately powered randomized controlled trials to evaluate the efficacy, safety, and long-term clinical benefits of dietary patterns, antioxidant interventions, ketogenic diets, caloric restriction, and fasting approaches across different tumor types and treatment settings. Such studies should also incorporate biomarkers of metabolic status, oxidative stress, and nutritional status to facilitate the implementation of precision nutrition strategies and enable the safe integration of these dietary interventions into routine oncology care [69,131].

This review has several limitations that should be acknowledged. As a narrative review, article selection was not based on a predefined systematic methodology, and study inclusion was therefore influenced, to some extent, by the authors’ judgment, which may have introduced selection bias. In addition, the non-systematic search strategy may have resulted in the omission of relevant studies, particularly those not indexed in the selected databases. Nevertheless, this approach enabled a more comprehensive and critical appraisal of the available evidence, allowing the integration of findings from diverse sources and the development of practical recommendations based on the most relevant literature. Future systematic reviews and meta-analyses are needed to confirm and expand upon the conclusions presented in this review.

## Figures and Tables

**Table 1:** Dietary approaches in cancer prevention.

Tumor Type	Dietary Intervention/Exposure	Study Design	Sample Size	Outcomes	Reference
Colorectal cancer	Foods rich in chalcones.	Narrative review	N/A	Dietary chalcones showed promising chemopreventive and anticancer activity in experimental models, but no clinical intervention studies in colorectal cancer were available.	[10]
Colorectal cancer	Low meat intake (reduced red and processed meat consumption)	Narrative review	N/A	High consumption of red and processed meat is consistently associated with an increased risk of colorectal cancer, supporting recommendations to limit meat intake as a preventive dietary strategy.	[11]
Colorectal cancer	Healthy dietary patterns (high fiber, whole grains, fruits and vegetables; low red and processed meat)	Narrative review	N/A	Higher intakes of dietary fiber, whole grains, fruits, vegetables, calcium, and dairy products were associated with a reduced risk of colorectal cancer, whereas high consumption of red and processed meat was associated with an increased risk.	[12]
Colorectal cancer	Healthy dietary patterns (high intake of whole grains, dairy products, dietary fiber and low intake of red and processed meat)	Systematic review and dose–response meta-analysis	N/A	Higher intakes of whole grains, dairy products, dietary fiber, and calcium were associated with a lower risk of colorectal cancer, whereas greater consumption of red and processed meat and alcohol was associated with an increased risk.	[4]
Colorectal cancer	Adherence to nutrition-based cancer prevention guidelines (high intake of plant-based foods and whole grains, limited red and processed meat, alcohol, and energy-dense foods)	Multicenter case–control study	5852 colorectal cancer cases and 5188 controls	Greater adherence to the World Cancer Research Fund/American Institute for Cancer Research (WCRF/AICR) nutrition-based cancer prevention guidelines was associated with a significantly lower risk of colorectal cancer.	[13]
Colorectal cancer	Exercise and healthy dietary patterns	Commentary/Perspective	N/A	Physical activity and healthy dietary patterns may enhance antitumor immune responses, reduce inflammation, and improve colorectal cancer survival, supporting the integration of lifestyle interventions into survivorship care.	[14]
Colorectal cancer	Probiotics	Narrative review	N/A	Probiotics may contribute to colorectal cancer prevention and support treatment by modulating the gut microbiota, reducing intestinal inflammation, strengthening epithelial barrier integrity, enhancing immune responses, and producing anticarcinogenic metabolites.	[15]
Breast cancer	Adherence to nutrition-based cancer prevention guidelines (high intake of plant-based foods and whole grains, limited red and processed meat, alcohol, and energy-dense foods)	Multicenter case–control study	1.343 breast cancer cases and 3.431 controls	Greater adherence to the World Cancer Research Fund/American Institute for Cancer Research (WCRF/AICR) nutrition-based cancer prevention guidelines was associated with a 15% lower risk of breast cancer for each one-point increase in the adherence score, with the strongest association observed for postmenopausal breast cancer.	[13]
Breast cancer	Vitamins and dietary micronutrients (vitamin D, folate, vitamin B6, β-carotene, curcumin, piperine, sulforaphane, indole-3-carbinol, quercetin, EGCG, omega-3 fatty acids)	Narrative review	N/A	Higher intakes of selected vitamins and dietary micronutrients were associated with a lower risk of breast cancer and recurrence through antiproliferative, anti-inflammatory, pro-apoptotic, and antiangiogenic mechanisms.	[16]
Breast cancer	Omega-3 fatty acids (EPA and DHA)	Narrative review	N/A	Higher intake and blood levels of EPA and DHA were associated with a lower risk of breast cancer in several observational studies. Omega-3 fatty acids may reduce inflammation, modulate growth factor signaling, and improve survivorship by alleviating treatment-related toxicities.	[17]
Breast cancer	Healthy dietary patterns and weight management (Mediterranean diet, fruits and vegetables, dietary fiber, reduced saturated fat, maintenance of healthy body weight)	Narrative review	N/A	Obesity and unhealthy dietary patterns were associated with an increased risk of postmenopausal breast cancer, whereas maintaining a healthy body weight and consuming a diet rich in plant-based foods may reduce breast cancer risk through improvements in metabolic, inflammatory, and hormonal pathways.	[18]
Breast cancer	Healthy diet, weight management, physical activity, and limited alcohol consumption	Narrative review	N/A	Maintaining a healthy body weight, engaging in regular physical activity, limiting alcohol intake, and following a healthy dietary pattern may substantially reduce breast cancer risk, particularly after menopause, and improve long-term survivorship outcomes.	[19]
Breast cancer	Mediterranean Diet	Systematic review and meta-analysis	N/A	greater adherence to the Mediterranean diet was associated with a significantly lower risk of breast cancer, particularly estrogen receptor-negative tumors, supporting the protective role of a dietary pattern rich in fruits, vegetables, whole grains, legumes, fish, and olive oil.	[20]
Breast cancer	Dietary patterns and specific food groups	Prospective cohort study (UK Women’s Cohort Study)	35,372 women (including 1822 incident breast cancer cases during follow-up)	Higher consumption of processed and red meat was associated with an increased risk of postmenopausal breast cancer, whereas greater intake of grapes and dried fruits was associated with a lower risk of breast cancer in specific subgroups.	[21]
Breast cancer	Dietary patterns, body weight management, and menopause-related nutrition	Narrative review	N/A	Maintaining a healthy body weight, consuming a diet rich in fruits, vegetables, whole grains, and dietary fiber, limiting red and processed meat and alcohol intake, and adopting healthy lifestyle habits may reduce breast cancer risk, particularly after menopause	[22]
Lung cancer	High fruit and vegetable intake	Systematic review and meta-analysis of prospective cohort studies	N/A	Higher fruit intake was associated with a significantly lower risk of lung cancer, particularly among current and former smokers. Higher vegetable intake was also associated with a reduced lung cancer risk, although the association was weaker.	[23]
Lung cancer	Mediterranean diet and dietary inflammatory index	Prospective cohort study	35,303 participants (Melbourne Collaborative Cohort Study; 403 incident lung cancer cases)	Greater adherence to the Mediterranean diet was associated with a lower risk of lung cancer, whereas a more pro-inflammatory diet, reflected by a higher Dietary Inflammatory Index score, was associated with an increased risk, particularly among current and former smokers	[24]
Lung cancer	Dietary vitamin A and β-carotene intake	Systematic review and meta-analysis	19 prospective studies	Higher dietary intakes of vitamin A and β-carotene were associated with a significantly lower risk of lung cancer, with the protective effect being more evident among current smokers. These findings support increasing the consumption of vitamin A- and β-carotene-rich foods as a potential strategy for lung cancer prevention.	[25]
Lung cancer	Healthy dietary pattern	Systematic review and meta-analysis	18 observational studies (8 cohort and 10 case–control studies)	Greater adherence to a healthy dietary pattern characterized by high consumption of fruits, vegetables, whole grains, fish, and poultry and low consumption of red and processed meat was associated with a significantly lower risk of lung cancer.	[26]
Lung cancer	Dietary cholesterol intake	Systematic review and meta-analysis	11 observational studies (8 case–control and 3 cohort studies)	Higher dietary cholesterol intake was associated with a significantly increased risk of lung cancer, particularly in case–control studies.	[27]
Lung cancer	Mediterranean diet and a posteriori derived dietary patterns	Case–control study	439 lung cancer cases and 844 controls	Greater adherence to the Mediterranean diet was associated with a significantly lower risk of lung cancer. Healthy dietary patterns rich in vegetables, fruits, whole grains, and fish were inversely associated with lung cancer risk, whereas unhealthy dietary patterns characterized by processed foods and red meat were associated with an increased risk	[28]
Lung cancer	Fruit and vegetable intake	Systematic review and meta-analysis	32 prospective studies	Higher consumption of fruits and vegetables was associated with a significantly lower risk of lung cancer. A dose–response relationship was observed, with increasing fruit intake showing the strongest inverse association, particularly among current smokers.	[29]
Ovarian cancer	Dairy products, calcium, and vitamin D intake	Population-based case–control study	490 ovarian cancer cases and 656 controls (African American women)	Higher dietary calcium intake was associated with a significantly lower risk of ovarian cancer, whereas no significant associations were observed for total dairy consumption or vitamin D intake.	[30]
Ovarian cancer	Modifiable lifestyle factors (healthy diet, body weight, physical activity, smoking, alcohol)	Narrative review	N/A	Maintaining a healthy body weight, following a healthy dietary pattern, engaging in regular physical activity, and avoiding smoking may contribute to reducing the risk of ovarian cancer,	[31]
Ovarian cancer	Dietary patterns and specific food groups	Prospective cohort study (UK Women’s Cohort Study)	35.372 women (including 337 incident ovarian cancer cases during follow-up)	No significant associations were observed between most dietary patterns or food groups and ovarian cancer risk. However, higher mushroom consumption was associated with a lower risk of ovarian cancer, while no consistent associations were found for red meat, fruits, vegetables, or dairy products	[21]
Ovarian cancer	Healthy dietary patterns, body weight management, and menopause-related nutrition	Narrative review	N/A	Maintaining a healthy body weight, consuming a diet rich in fruits, vegetables, whole grains, and dietary fiber, and limiting red and processed meat may contribute to reducing ovarian cancer risk.	[22]
Ovarian cancer	Dietary and nutrient intake (Mediterranean diet, fruits and vegetables, soy isoflavones, vitamins, vitamin D, tea, coffee, dairy products, dietary fat)	Narrative review	N/A	Greater adherence to healthy dietary patterns, particularly the Mediterranean diet and diets rich in fruits, vegetables, and soy products, was associated with a lower risk of ovarian cancer, whereas high-fat diets may increase risk. Evidence for individual nutrients remains inconsistent and further prospective studies are needed.	[32]
Prostate cancer	Adherence to WCRF/AICR cancer prevention dietary guidelines	Case–control study	1.555 cases; 3076 controls	Greater adherence to cancer prevention recommendations was associated with a lower risk of prostate cancer.	[13]
Prostate cancer	Dairy products, saturated fat and calcium intake	Prospective cohort study	43.435	High dairy and calcium intake were evaluated in relation to prostate cancer risk.	[33]
Prostate cancer	Higher plasma lycopene (reflecting tomato-rich diet)	Nested case–control study	578 cases; 1.294 controls	Higher plasma lycopene concentrations were associated with a lower prostate cancer risk.	[34]
Prostate cancer	Dietary carotenoids, retinol and tocopherols	Pooled analysis of 15 prospective studies	11.239	No consistent association between carotenoids, retinol, tocopherols and prostate cancer risk	[35]
Prostate cancer	Dietary fatty acid intake	Cross-sectional study	69	Dietary fatty acid intake was associated with biomarkers of DNA damage in men with prostate cancer	[36]
Prostate cancer	Dietary fiber intake	Pooled analysis of 15 prospective cohort studies	>840,000 men; 36,661 cases	Higher dietary fiber intake was associated with a lower risk of advanced and aggressive prostate cancer.	[37]
Prostate cancer	Vitamin E (400 IU/day) ± selenium supplementation	Randomized controlled prevention trial	35.533	Vitamin E supplementation increased prostate cancer risk, with no evidence of preventive benefit.	[38]

**Table 2:** Dietary approaches in cancer treatment.

Cancer Type	Dietary Intervention	Study Design	Sample Size	Outcomes	Reference
Stage IV recurrent colon cancer	Modified medium-chain triglyceride (MCT) ketogenic diet + chemotherapy	Prospective clinical study (conference abstract)	10	Combination therapy showed higher response rate, disease control rate and conversion surgery rate than chemotherapy alone. Authors suggested a ketogenic diet may be a supportive treatment, but evidence is preliminary ^1^.	[109]
Non-metastatic rectal cancer	Ketogenic diet based on natural foods, supplemented with essential amino acids, during neoadjuvant radio-chemotherapy	Prospective, controlled, non-randomized clinical trial (KETOCOMP)	41	The ketogenic diet was feasible and safe, significantly reducing body weight and fat mass while preserving skeletal muscle mass. Patients receiving the ketogenic diet showed a trend toward improved pathological tumor regression after neoadjuvant radio-chemotherapy ^1^.	[110]
HER2-negative stage II–III breast cancer	STF (24 h before and 24 h after each TAC chemotherapy cycle)	Randomized pilot study	13	STF was feasible and well tolerated. Patients in the STF group had significantly higher erythrocyte and platelet counts after chemotherapy and reduced chemotherapy-induced DNA damage in peripheral blood mononuclear cells, suggesting protection of normal cells without compromising treatment ^2^.	[111]
Breast cancer	Fish oil (omega-3 fatty acid supplements) and other dietary supplements during chemotherapy	Prospective observational cohort nested within the SWOG S0221 phase III randomized trial	1.134	Fish oil use was not associated with significantly worse disease-free survival or overall survival. In contrast, antioxidant supplements, vitamin B12 and iron supplementation were associated with poorer clinical outcomes ^3^.	[112]
ER+/PR±/HER2− breast cancer receiving antiestrogenic therapy (with or without prior chemotherapy)	High-protein, calcium-rich diet with probiotics and prebiotics, alone or combined with isometric exercise for 1 year	Randomized controlled trial	165	Dietary intervention, particularly when combined with exercise, improved body composition, reduced fat mass, preserved lean body mass, and improved metabolic parameters during antiestrogenic treatment. No oncological outcomes (recurrence or survival) were assessed.	[113]
Breast and ovarian cancer	Short-term fasting (60 h around chemotherapy)	Randomized crossover pilot study	34	Short-term fasting was feasible and safe, reduced deterioration in quality of life and fatigue during chemotherapy, and did not interfere with treatment delivery. No conclusions on tumor response or survival could be drawn ^2^.	[114]
Early-stage breast cancer	Individualized dietary counseling combined with adapted physical activity during adjuvant chemotherapy and radiotherapy	Randomized controlled trial	143	The intervention improved physical activity levels, limited declines in quality of life and physical functioning, and helped maintain healthier lifestyle behaviors. It did not evaluate tumor response or survival outcomes.	[115]
Early-stage breast cancer receiving chemotherapy	Plant-forward healthy diet counseling combined with structured physical activity and behavioral coaching	Randomized controlled trial	173	The lifestyle intervention significantly improved diet quality, physical activity, and adherence to healthy lifestyle recommendations during chemotherapy, but did not assess cancer recurrence or survival outcomes.	[116]
Early-stage breast cancer	Prudent dietary pattern (high intakes of fruits, vegetables, whole grains, and poultry	Prospective cohort study (Life After Cancer Epidemiology [LACE] Study)	1.901	A prudent dietary pattern was not associated with a reduced risk of breast cancer recurrence or all-cause mortality. In contrast, a Western dietary pattern was associated with increased mortality, particularly from non-breast-cancer causes.	[117]
Locally recurrent or metastatic HER2-negative breast cancer	Ketogenic diet combined with irinotecan	Randomized controlled trial protocol	518	Primary outcomes: chemotherapy sensitivity and objective response rate; secondary outcomes: progression-free survival, overall survival, quality of life, adverse events, and cost-effectiveness ^1^.	[118]
Stage III epithelial ovarian cancer	Calorie-restricted “weight change therapy” combined with neoadjuvant carboplatin (Kemocarb) and paclitaxel	Case report	1	The patient experienced marked weight loss, improvement in performance status, and underwent interval surgery after neoadjuvant chemotherapy. Because this is a single case report, no conclusions regarding the efficacy of the dietary intervention can be drawn ^4^.	[119]
Ovarian and endometrial cancer	Ketogenic diet (70% fat, 25% protein, 5% carbohydrate) versus American Cancer Society diet	Randomized controlled trial	45	The ketogenic diet was feasible and safe, improved physical function and perceived energy, and reduced cravings for starchy foods and fast-food fats. No improvement in oncological outcomes was evaluated ^1^.	[120]
Advanced non-small cell lung cancer (NSCLC)	Fish oil supplementation (2.5 g/day EPA + DHA) during first-line chemotherapy	Prospective clinical trial with concurrent control group	46	Fish oil supplementation was associated with significantly higher chemotherapy response and clinical benefit rates, without increasing toxicity, suggesting improved efficacy of carboplatin-based chemotherapy ^3^.	[121]
Advanced non-small cell lung cancer (NSCLC)	Fish oil supplementation (2.2 g/day EPA) during carboplatin-based chemotherapy	Prospective clinical study with concurrent control group	40	Fish oil supplementation attenuated weight loss and preserved skeletal muscle mass during chemotherapy compared with standard care, suggesting improved nutritional status without increasing treatment-related toxicity ^3^.	[122]
Stage III non-small cell lung cancer (NSCLC)	Oral nutritional supplement containing 2.02 g EPA + 0.92 g DHA/day during multimodality treatment	Double-blind randomized controlled trial	40	n-3 PUFA supplementation significantly improved quality of life, physical and cognitive functioning, Karnofsky Performance Status, and physical activity compared with an isocaloric control, without significantly affecting handgrip strength ^5^.	[123]
Advanced non-small cell lung cancer (NSCLC)	Early intensive nutritional intervention with individualized dietary counseling and oral nutritional supplements during chemotherapy with guideline-based antiemetic therapy	Prospective clinical study with retrospective matched controls	10 intervention; 38 retrospective controls	Early nutritional intervention prevented significant weight loss during cytotoxic chemotherapy and increased the proportion of patients who gained weight compared with controls ^6^.	[124]
Advanced non-small cell lung cancer (NSCLC) and pancreatic cancer	NEXTAC program: individualized nutritional counseling, branched-chain amino acid–enriched oral nutritional supplement, home-based resistance exercise, and physical activity counseling during first-line chemotherapy	Multicenter randomized phase II study protocol	130	Study designed to evaluate whether the multimodal NEXTAC program improves disability-free survival, nutritional status, physical function, muscle mass, and quality of life in elderly patients receiving first-line chemotherapy ^6^.	[125]
Advanced non-small cell lung cancer (stage IIIB–IV)	AferBio, a fermented nutritional supplement rich in β-glucans, amino acids, vitamin B12 and selenium, administered during second-line palliative chemotherapy	Phase II, double-blind, randomized, placebo-controlled study protocol	94 planned	The trial was designed to evaluate whether AferBio^®^ improves health-related quality of life and reduces chemotherapy-related toxicity and infectious complications. This publication reports only the study protocol; no efficacy or safety results were available ^6^.	[126]
Metastatic non-small cell lung cancer (NSCLC)	Ketogenic diet combined with metabolically supported chemotherapy (weekly carboplatin/paclitaxel), hyperthermia, and hyperbaric oxygen therapy	Retrospective single-center feasibility study	44	The multimodal approach was feasible and well tolerated, with an objective response rate of 61.4%, median progression-free survival of 41.0 months, and median overall survival of 42.9 months ^1^.	[127]
Newly diagnosed glioblastoma	Ketogenic diet as an adjunct to standard chemoradiotherapy with temozolomide	Randomized pilot study protocol	12	The study was designed to assess the feasibility, safety, tolerability, and quality-of-life impact of two ketogenic diet regimens during standard chemoradiotherapy. No clinical results were reported in this publication ^1^.	[128]
Newly diagnosed glioblastoma	Ketogenic diet (3:1 ratio) during standard radiotherapy plus temozolomide chemotherapy	Prospective pilot feasibility study	9	The ketogenic diet was feasible and generally well tolerated during concurrent chemoradiotherapy. Most patients achieved nutritional ketosis, and no unexpected severe diet-related toxicities were reported ^1^.	[129]
Glioblastoma	MAD during radiotherapy and temozolomide chemotherapy	Retrospective feasibility study	29	The MAD was feasible and safe, with 100% of patients achieving ketosis and no serious diet-related adverse events. Among the glioblastoma patients, 58% developed pseudoprogression after radiotherapy plus temozolomide, suggesting a potential radiosensitizing effect ^1^.	[130]

Notes: (1) Although the KD has shown promising results in selected studies, current evidence is insufficient to support its routine use in cancer patients. Particular caution is warranted in patients at risk of malnutrition or cancer cachexia, with careful attention to patient safety and nutritional status; (2) however, fasting diets should only be considered after careful nutritional assessment to minimize the risk of malnutrition, excessive weight loss, and impaired treatment tolerance.; (3) although fish oil supplementation appears to be safe, current evidence is insufficient to support its routine use in cancer patients. Supplementation should therefore be individualized and considered with attention to patient safety under appropriate medical and nutritional supervision; (4) although CR has shown promising biological effects, current clinical evidence remains limited. Its use should be approached cautiously, particularly in patients at risk of malnutrition or cancer cachexia, with careful attention to patient safety and nutritional status; (5) although PUFA supplementation appears to be safe, current evidence is insufficient to support its routine use in cancer patients. Its use should be individualized with careful attention to patient safety and nutritional status; (6) nutritional supplementation has shown promising effects in selected studies, however current evidence is insufficient to support its routine use in cancer patients. Supplementation should be individualized with careful attention to patient safety. Abbreviations: pt, patients; KD, ketogenic diet; MAD, modified Atkins diet; MD, Mediterranean diet; STF, short-term fasting, PUFA, Polyunsaturated Fatty Acids.

**Table 3:** Summary of clinical evidence on nutritional interventions in cancer.

Category	Study	Cancer Type	Nutritional Intervention	Study Design	N	Main Findings	Main Limitations
Vitamin and antioxidant supplementation	ATBC Study Group, 1994 [245]	Lung cancer prevention	α-Tocopherol, β-carotene	Randomized DB placebo-controlled factorial trial	29.133	β-Carotene increased lung cancer incidence and mortality.	Male smokers only; prevention trial.
Vitamin and antioxidant supplementation	Bairati et al., 2006 [44]	Head and neck cancer	Antioxidant vitamin supplementation (α-tocopherol and β-carotene) during radiotherapy	Multicenter randomized, double-blind, placebo-controlled trial	540 randomized	Antioxidant supplementation reduced acute radiotherapy-related adverse effects but was associated with a significantly higher rate of second primary cancers, particularly among smokers, without improving overall survival.	Conducted in head and neck cancer only; adverse effects mainly observed in smokers; findings may not be generalizable to other tumor types or treatment settings.
Vitamin and antioxidant supplementation	Klein et al. (SELECT), 2011 [38]	Prostate cancer prevention	Vitamin E, selenium	Multicenter RCT	35.533	Vitamin E increased prostate cancer risk; selenium no benefit.	Healthy men; prevention setting.
Vitamin and antioxidant supplementation	Lance et al., 2017 [169]	Colorectal adenoma prevention	Selenium, vitamin E	Secondary analysis of SELECT	6.546	No reduction in adenoma risk.	Secondary analysis; prevention.
Vitamin and antioxidant supplementation	Ambrosone et al., 2020 [112]	Breast cancer	Dietary supplements during chemotherapy	Prospective cohort nested within SWOG S0221	1.134	Antioxidants associated with increased recurrence; B12/iron with poorer DFS.	Observational; supplement use not randomized.
Ketogenic diet/carbohydrate restriction	Klement & Sweeney, 2016 [188]	Breast, prostate, rectal and lung cancer	Ketogenic diet	Prospective pilot study	6	Feasible; preserved lean body mass.	Very small sample; no control.
Ketogenic diet/carbohydrate restriction	Zahra et al., 2017 [190]	Lung and pancreatic cancer	Ketogenic diet during chemoradiotherapy	Phase I clinical trial	9	Feasible in subset; safety evaluated.	Poor adherence; heterogeneous tumors; no efficacy endpoints.
Ketogenic diet/carbohydrate restriction	Cohen et al., 2018 [120]	Ovarian/endometrial cancer	Ketogenic diet	RCT	43	Improved physical function, energy and body composition.	Small sample; short follow-up.
Ketogenic diet/carbohydrate restriction	Freedland et al. (CAPS2), 2020 [153]	Biochemically recurrent prostate cancer	Low-carbohydrate diet	RCT	45	Metabolic benefits; adjusted analyses suggested longer PSA doubling time.	Primary endpoint not met; small sample.
Ketogenic diet/carbohydrate restriction	Woodhouse et al., 2019 [130]	glioma	Modified Atkins ketogenic diet	Prospective feasibility study	8	Diet feasible during radiotherapy; ketosis achieved in most patients	Small sample; no control group
Ketogenic diet/carbohydrate restriction	Rieger et al., 2014 (ERGO) [212]	Recurrent glioblastoma	Ketogenic diet	Pilot clinical study	20	Ketogenic diet feasible and safe; limited evidence of antitumor efficacy	Non-randomized; small sample
Ketogenic diet/carbohydrate restriction	Champ et al., 2014 [216]	glioblastoma	Ketogenic diet	Prospective pilot study	6	Ketosis achieved; treatment feasible during radiotherapy	Very small sample
Ketogenic diet/carbohydrate restriction	Chi et al., 2022 [154]	Prostate cancer	Low-carbohydrate diet	Secondary analysis of CAPS2 RCT	45	Higher ketone levels associated with slower PSA doubling time	Secondary analysis; surrogate endpoint
Fasting	Fay-Watt et al. 2023 [155]	Prostate cancer	Fasting-mimicking diet (FMD)	Prospective pilot implementation study	35	Three monthly FMD cycles were feasible and safe, with little or no toxicity, high adherence among completers, and improvements in several metabolic parameters	Small cohort; non-randomized; no control group; efficacy on cancer outcomes not established
Short-term fasting	de Groot et al., 2015 [111]	HER2-negative breast cancer	Short-term fasting	Randomized pilot trial	13	Reduced chemotherapy-induced DNA damage.	Pilot; small sample.
Short-term fasting	Bauersfeld et al., 2018 [114]	Breast/ovarian cancer	Short-term fasting	Randomized cross-over pilot trial	34	Improved quality of life and fatigue.	Small sample; no survival endpoints.
Omega-3 and oral nutritional supplementation	Murphy et al., 2011 [121]	Advanced NSCLC	Fish oil (EPA/DHA)	Prospective non-randomized intervention	46	Higher chemotherapy response; better weight/muscle preservation.	Non-randomized; small sample.
Omega-3 and oral nutritional supplementation	van der Meij et al., 2012 [123]	Lung cancer	n-3 PUFA oral supplements	RCT	40	Improved QoL and nutritional status.	Small sample; no survival endpoints.
Nutritional counseling	Tanaka et al., 2018 [124]	Advanced lung cancer	Dietary counseling + antiemetic treatment	Prospective clinical study	48	Reduced weight loss and improved nutritional status.	Non-randomized; no survival outcomes.
Nutritional counseling	Carayol et al., 2019 [115]	Breast cancer	Diet counselling + physical activity	RCT	143	Improved lifestyle outcomes during adjuvant therapy	Multifactorial intervention
Nutritional counseling	Bourdel-Marchasson et al., 2014 [157]	Older patients with cancer	Individualized nutritional counseling	RCT	336	Improved nutritional status but not survival or treatment completion.	Heterogeneous cancers.

## Data Availability

No new data were created or analyzed in this study. Data sharing is not applicable to this article.

## Abbreviations

The following abbreviations are used in this manuscript:

WCRF	World Cancer Research Fund International
AICR	American Institute for Cancer Research
CRC	Colorectal cancer
SCFAs	Short-chain fatty acids
VDR	Vitamin D receptor
PUFAs	Polynsaturated fatty acids
MUFAs	Monounsaturated fatty acids
BMI	Body mass index
NOCs	N-nitroso compounds
MD	Mediterranean diet
EGCG	Epigallocatechin gallate
ALA	Alpha-linoleum acid
EPA	Eicosapentaenoic acid
DHA	Docosahexaenoic acid
IGF-1	Insulin-like growth factor-1
VEGF	vascular endothelial growth factor
GnRH	Gonadotropin-releasing hormone
FSH	Follicle-stimulating hormone
LH	Luteinizing hormones
LDL	Low-density lipoprotein
FMT	Fecal microbiome transplantation
LDL-C	LDL cholesterol
RCTs	Randomized controlled trials
LCFAs	Long-chain fatty acids
CR	Caloric restrictions
STF	Short-term fasting
CNS	Central nervous system
*β*HB	*β*-hydroxybutyrate
MCTs	Medium chain triglycerides
PTX	Paclitaxel
PTH	Parathyroid hormone
KD	Ketogenic diet
NAPH	Nicotinamide adenine dinucleotide phosphate
SIRT 1	sirtuin 1
NRF-2	nuclear factor erythroid 2-related factor 2
PI3K	phosphoinositol 3-kinase
SAM	S-adenosylmethionine
